# Childhood, adolescent, and adulthood adiposity are associated with risk of PCOS: a Mendelian randomization study with meta-analysis

**DOI:** 10.1093/humrep/dead053

**Published:** 2023-04-04

**Authors:** Laurence J Dobbie, Bradley Pittam, Sizheng Steven Zhao, Uazman Alam, Theresa J Hydes, Thomas M Barber, Daniel J Cuthbertson

**Affiliations:** Department of Cardiovascular and Metabolic Medicine, Institute of Life Course and Medical Sciences, University of Liverpool, Liverpool, UK; University Hospital Aintree, Liverpool University Hospitals NHS Foundation Trust, Liverpool, UK; Manchester University Hospital NHS Foundation Trust, Manchester, Greater Manchester, UK; Centre for Epidemiology Versus Arthritis, Division of Musculoskeletal and Dermatological Science, School of Biological Sciences, Faculty of Biological Medicine and Health, The University of Manchester, Manchester Academic Health Science Centre, Manchester, UK; Department of Cardiovascular and Metabolic Medicine, Institute of Life Course and Medical Sciences, University of Liverpool, Liverpool, UK; University Hospital Aintree, Liverpool University Hospitals NHS Foundation Trust, Liverpool, UK; Department of Cardiovascular and Metabolic Medicine, Institute of Life Course and Medical Sciences, University of Liverpool, Liverpool, UK; University Hospital Aintree, Liverpool University Hospitals NHS Foundation Trust, Liverpool, UK; Warwickshire Institute for the Study of Diabetes, Endocrinology and Metabolism, University Hospitals Coventry and Warwickshire, Coventry, UK; Warwick Medical School, University of Warwick, Coventry, UK; Department of Cardiovascular and Metabolic Medicine, Institute of Life Course and Medical Sciences, University of Liverpool, Liverpool, UK; University Hospital Aintree, Liverpool University Hospitals NHS Foundation Trust, Liverpool, UK

**Keywords:** insulin resistance, hyperinsulinaemia, polycystic ovary syndrome, obesity, Mendelian randomization, epidemiology

## Abstract

**STUDY QUESTION:**

What is the influence of body composition during childhood, adolescence, and adulthood, as well as metabolic parameters, on incident polycystic ovary syndrome (PCOS)?

**SUMMARY ANSWER:**

Excess body fat, even during childhood/adolescence, and metabolic parameters, suggestive of hyperinsulinaemia/insulin resistance, significantly impact the risk of PCOS in a linear fashion.

**WHAT IS KNOWN ALREADY:**

Observational and Mendelian randomization (MR) data have demonstrated an association between adulthood overweight/obesity and development of PCOS. However, the contribution of body composition in childhood/adolescence to incident PCOS is unclear, as is the influence of childhood overweight/obesity.

**STUDY DESIGN, SIZE, DURATION:**

We conducted a systematic review and meta-analysis and integrated our results with a previously published systematic review. Two blinded investigators screened abstracts published between November 2010 and May 2021. Furthermore, we incorporated summary statistics from genome-wide association study (GWAS) data in subjects of European ancestry. Adult overweight was defined as BMI ≥ 25 kg/m^2^ and obesity as BMI ≥ 30 kg/m^2^; in Asian subjects, overweight was defined as BMI ≥ 23 kg/m^2^ and obesity as BMI ≥ 25 kg/m^2^.

**PARTICIPANTS/MATERIALS, SETTING, METHODS:**

We utilized meta-analysis and MR together to allow synthesis of genetic and observational data. For the systematic review, the search revealed 71 studies, of which 63 were included in meta-analysis by calculating odds ratios (ORs) using the random-effects model. Furthermore, we conducted a two-sample MR study of GWAS data to determine the impact of childhood and adult body size (defined categorically by BMI and childhood body size proportions), abnormal body composition and metabolic parameters (higher fasting serum insulin or lower sex hormone-binding globulin (SHBG) concentration) on the odds of incident PCOS via the inverse-variance weighted method.

**MAIN RESULTS AND THE ROLE OF CHANCE:**

Significant associations were shown between body composition and PCOS incidence. From the systematic review/meta-analysis, women with overweight (OR 3.80, 2.87–5.03), obesity (OR 4.99, 3.74–6.67), and central obesity (OR 2.93, 2.08–4.12) had increased odds of PCOS. For adolescents with overweight and/or obesity, the PCOS odds were greater than for adults. From MR, for every standard deviation increase in BMI (4.8 kg/m^2^), the odds of PCOS increased by 2.76 (2.27–3.35). Childhood body size had an independent effect on PCOS odds after adjusting for adult body size (OR: 2.56, 1.57–4.20). Genetically determined body fat percentage (OR 3.05, 2.24–4.15), whole body fat mass (OR 2.53, 2.04–3.14), fasting serum insulin (OR 6.98, 2.02–24.13), and SHBG concentration (OR 0.74, 0.64–0.87) were all significantly associated with PCOS in a linear relation.

**LIMITATIONS, REASONS FOR CAUTION:**

The meta-analysis included studies which were cross-sectional and retrospective, limiting our ability to determine causality. MR was limited by interrogating subjects only of European ancestry and including cases classified by either self-diagnosis or diagnostic criteria.

**WIDER IMPLICATIONS OF THE FINDINGS:**

Our study demonstrates for the first time a critical role of the impact of excess childhood/adolescent adiposity on the pathophysiology of adult PCOS. Our results, driven by genetically determined childhood/adolescent body composition, higher BMI, hyperinsulinaemia, and lower SHBG, clearly favour obesity driving the metabolic, but not reproductive, PCOS phenotype. Overall, effective weight maintenance, even from the early years, is likely to reduce the risk of this reproductive endocrine disorder.

**STUDY FUNDING/COMPETING INTEREST(S):**

S.S.Z. was funded by a National Institute for Health and Care Research (NIHR) Academic Clinical Lectureship. U.A. is chair of the NIHR Steering Committee Trial—CASSANDRA-DN. No other authors declare any sources of funding or relevant conflicts of interest. The authors declare that the research was conducted in the absence of any commercial or financial relations that could be construed as a potential conflict of interest.

**TRIAL REGISTRATION NUMBER:**

N/A.

## Introduction

Polycystic ovary syndrome (PCOS) is a highly prevalent disease, affecting 6–20% of women (Diamanti-Kandarakis *et al.*, 1999; Asunción *et al.*, 2000; Azziz *et al.*, 2004b), characterized by clinical and/or biochemical androgen excess, menstrual abnormalities, and sub-/infertility (Hull, 1987; Balen *et al.*, 1995b; Boomsma *et al.*, 2006; Kjerulff *et al.*, 2011; Legro *et al.*, 2013). It has a significant impact on quality of life (Barnard *et al.*, 2007) and is commonly associated with metabolic abnormalities (including features of the metabolic syndrome, type 2 diabetes, and non-alcoholic fatty liver disease) (Azziz *et al.*, 2009; Escobar-Morreale, 2018; Sanchez-Garrido and Tena-Sempere, 2020; Moghetti and Tosi, 2021) and cardiovascular disease (Zhang *et al.*, 2020). Lifestyle interventions (diet and exercise) (Palomba *et al.*, 2015; Kim *et al.*, 2020) represent the first-line of treatment although therapeutic approaches include targeting insulin resistance (metformin, glitazones), androgen excess (oral contraceptive pill), weight loss (glucagon-like Peptide 1 (GLP1)-receptor agonists) (Lamos *et al.*, 2017; Han *et al.*, 2019; Cena *et al.*, 2020), and fertility (i.e. clomiphene citrate) (Escobar-Morreale, 2018; Pasquali, 2018).

Obesity/overweight are associated with PCOS, with observational evidence reporting a 33–88% prevalence of overweight/obesity in women with PCOS (Balen *et al.*, 1995a; Gambineri *et al.*, 2002; Barber *et al.*, 2006; Lim *et al.*, 2012; Ollila *et al.*, 2016; Aarestrup *et al.*, 2021). The huge variation in prevalence likely reflects differences in study settings (e.g. community, endocrine or infertility clinics) and differences in the PCOS diagnostic criteria applied. Central obesity is also crucial, given its association with hyperandrogenism and insulin resistance (Ollila *et al.*, 2016). A previous meta-analysis in 2012 demonstrated that women with PCOS had an increased prevalence of overweight (relative risk, RR: 1.95, 95% CI: 1.52, 2.50) and obesity (RR: 2.77, 95% CI: 1.88, 4.10) compared with controls (Lim *et al.*, 2012). This study, however, was limited by including a small number of studies of adolescents. This meant subgroup analysis was non-significant when comparing the impact of overweight on PCOS prevalence in adults and adolescents (RR, Adults 1.92, 95% CI: 1.48–2.48, Adolescents: 2.25, 95% CI: 0.42–11.98). In addition, these observational data are susceptible to confounding and, more importantly, reverse causation, with the concern that PCOS pathophysiology may in turn influence body size. Therefore, it is crucial to determine whether overweight and obesity are causal, given that BMI is a modifiable risk factor that is amenable to lifestyle (diet and exercise) (Dobbie *et al.*, 2021; Hwalla and Jaafar, 2021), pharmacological (i.e. GLP-1 receptor agonists) (Vilsbøll *et al.*, 2012; Wilding *et al.*, 2021) or (metabolic) surgical intervention (Gloy *et al.*, 2013; O’Brien *et al.*, 2019).

Metabolic abnormalities, including insulin resistance and reduced sex hormone-binding globulin (SHBG) concentrations, have been implicated in PCOS. SHBG is generated by the liver and as the primary protein that binds sex steroids (such as oestradiol and testosterone), reduced SHBG levels results in elevated biologically active free testosterone levels, and thus hyperandrogenaemia. Low serum SHBG levels are considered a biomarker of metabolic abnormalities and are associated with insulin resistance as hyperinsulinaemia inhibits hepatic SHBG production (Qu and Donnelly, 2020). Approximately 75% of people with PCOS have insulin resistance (Tosi *et al.*, 2017). Insulin resistance induces clinical features of PCOS; however, hyperandrogenaemia (from PCOS) also causes insulin resistance (Moghetti and Tosi, 2021). Current observational evidence lacks the ability to determine causality of insulin resistance on PCOS (Diamanti-Kandarakis and Dunaif, 2012). Genetic meta-analysis has demonstrated that eight or more polymorphisms of *SHBG* may predict increased PCOS risk (Li *et al.*, 2021). However, these are a lack of evidence evaluating the effect of hyperinsulinaemia, SHBG levels, or altered body composition on PCOS risk.

Mendelian randomization (MR) is an observational approach whereby genetic variants are used as instrumental variables to estimate the causal effect of an exposure (in this case, overweight/obesity) on an outcome (development of PCOS). Since variants are randomly allocated at conception, MR is less susceptible to reverse causation and confounding than other observational designs. Prior MR studies have shown BMI to have a causal association with PCOS risk, but not vice versa (Day *et al.*, 2015; Brower *et al.*, 2019; Zhao *et al.*, 2020; Yan *et al.*, 2021). Existing studies leave several unanswered questions. First, contemporaneous BMI provides insight only into adult determinants, whereas the relative importance of childhood or adult body sizes remains unexplored. Second, recognizing the limitations of the BMI as a metric of excess body weight and as an index for adiposity, capturing both lean and fat mass, we were interested in examining the role of central obesity (Shah and Braverman, 2012; Nuttall, 2015). Finally, given the association between metabolic parameters (i.e. hyperinsulinaemia and low SHBG levels) and PCOS we wanted to examine the effect of these on incident PCOS.

Our aim was to evaluate the evidence regarding the association of overweight, obesity, and central obesity with incident PCOS, updating the previous meta-analysis conducted by Lim *et al.* (2012). Subsequently, we sought to evaluate the causal impact of BMI on the development of PCOS by performing a series of two-sample MR studies to address the unresolved issues of the role of childhood and adult body size, metabolic parameters and the influence specifically of abnormal body composition.

## Materials and methods

### Systematic review and meta-analysis

We conducted a systematic review and meta-analysis interrogating how body composition during childhood, adolescence, and adulthood impacts PCOS risk. We utilized the systematic review methodology previously published by Lim *et al.* (2012). In total, 101 of the studies from this review concerned the prevalence of obesity in women with PCOS; these were excluded as they did not include a control group. Thirty-five of the studies from the previous systematic review (Lim *et al.*, 2012) were carried through to our meta-analysis.

#### Search strategy

We conducted a literature search based on the previous systematic review strategy (Supplementary Table SI). All articles published after and including November 2010 were screened up until May 2021 (Lim *et al.*, 2012). In addition to this, review articles were screened.

#### Selection criteria

To allow meta-analysis, we extracted the most consistently reported outcome(s).

#### Inclusion criteria

This systematic review considered the odds of PCOS in adolescents and adults with/without overweight, obesity, and central obesity. Studies in which women with PCOS were consecutively recruited or randomly sampled were included. For study inclusion, PCOS was defined according to the National Institutes of Health (NIH), ESHRE/American Society for Reproductive Medicine (ASRM) criteria (also termed Rotterdam criteria) or Androgen Excess-PCOS (AE-PCOS criteria) (Supplementary Table SII) (Wild *et al.*, 2010; Fauser *et al.*, 2012; Conway *et al.*, 2014). Studies comparing odds of PCOS in women with and without overweight/obesity were included if control subjects were not matched based on weight or BMI. Studies assessing odds of PCOS in women with/without central obesity were included if control subjects were not matched based on waist circumference (WC) or waist–hip ratio (WHR).

#### Exclusion criteria

We excluded studies where participants were selected by body weight, BMI, WC, or WHR.

#### Definitions

Body weight was analysed as a binary outcome (overweight/non-overweight; obesity/no obesity; central obesity/no central obesity). In addition, BMI cut-offs vary between different ethnicities (e.g. White Europeans versus South-East Asians), meaning meta-analysis of continuous BMI data would be challenging. Adult overweight was defined as BMI ≥ 25 kg/m^2^ and obesity as BMI ≥ 30 kg/m^2^ as per World Health Organization (WHO) criteria (World Health Organization, 2000). In studies of Asian subjects, overweight was BMI ≥ 23 kg/m^2^ and obesity was BMI ≥ 25 kg/m^2^ according to the International Obesity Task Force (Goda and Masuyama, 2016). For adolescents, age-gender-specific percentile BMI distributions were used to define overweight as the 85–95th percentile and obesity as the ≥95 percentile (Barlow, 2007). Central obesity was defined as a WC ≥80 cm (IDF, 2020), WHR >0.85 (World Health Organization, 2000), or WC >88 cm according to the Adult Treatment Panel III (Expert Panel on Detection and Treatment of High Blood Cholesterol in Adults, 2001). The central obesity definition was applied (Supplementary Table SIII) depending on that utilized in the included paper, as per the previous systematic review (Lim *et al.*, 2012).

#### Record screening

Two reviewers screened half of the studies identified (L.J.D. and B.P.) with each independently validating 10% of the others work. Discrepancies were resolved through discussion with co-authors.

#### Data extraction

General study characteristics (author, publication year, study location, study period, study design, number of women with and without PCOS), characteristics of the study population (recruitment source, sampling method, age, ethnicity), the definition of PCOS (NIH, ESHRE/ASRM, AE-PCOS), pre-existing medication use, physical activity and diet history, the definition of overweight/obesity (WHO, International Obesity Task Force (IOTF) and central obesity (International Diabetes Federation (IDF) WHO, Adult Treatment Panel III (ATP III)), measurements of height, weight, and WC, and the proportion of women with overweight, obesity, or central obesity were extracted from all included studies. Data were extracted in duplicate (L.J.D. and B.P.).

#### Quality assessment

Two reviewers (L.J.D., and D.J.C.) assessed risk of bias using the Newcastle-Ottawa Scale for all included studies.

#### Outcomes of interest

The primary endpoints were odds of PCOS in adolescent and adult women with/without overweight, obesity, and central obesity.

#### Meta-analysis

Data collected here was integrated with the meta-analysis by Lim *et al.* (2012). Our analytical technique differed, as we calculated odds ratio (OR) rather than RR. Random effects models were used to estimate the pooled prevalence, using the inverse variance weighting method. Heterogeneity of meta-analysis estimates was presented using the *I*^2^ statistic. Funnel plots were used to assess risk of publication bias. Sensitivity analysis assessed the impact of age (adult versus adolescence) and study type (Cross-sectional, Retrospective, Case-control, Cohort). Analyses were performed using R version 3.6.2 (R Foundation for Statistical Computing, Vienna, Austria) and the ‘meta’ and ‘metaphor’ packages.

### Mendelian randomization

#### Data sources

Data for BMI associated traits were obtained from genome-wide associations studies (GWAS), as summarized in Table I. We instrumented BMI using GWAS of both males and females, since preliminary analyses showed that sex-specific instruments did not provide meaningful difference to results. By contrast, SHBG instruments did provide different result; therefore, we chose variants from GWAS of female SHBG. PCOS data were obtained from a GWAS meta-analysis of 10 074 cases and 103 164 controls of European ancestry. PCOS was diagnosed according to NIH, Rotterdam criteria or self-diagnosis (Day *et al.*, 2018) (Supplementary Figs S1 and S2).

**Table 1:** Summary data of genetic instruments used for Mendelian randomization.

	Unit	Sample size (n=)	Number of SNPs	*R*2	*F*
**Childhood body size**	Thinner, About Average, Plumper	453 169	313	2.0%	30
**Adult body size**	BMI categorized into childhood body size proportions	453 169	580	2.9%	23
**BMI (kg/m^2^)**	4.8 kg/m^2^	806 834	543	5.7%	89
**Whole body fat mass (kg)**	9.6 kg	454 137	435	6.6%	58
**Body fat percentage (%)**	8.5%	454 633	395	5.3%	55
**Appendicular lean mass (kg)**	5.6 kg	450 243	690	10.4%	97
**Fasting insulin**	0.79 pmol/l	108 557	14	NA	NA
**Glycated haemoglobin**	6.7 mmol/mol	344 182	320	11.9%	146
**SHBG**	27.7 nmol/l	312 215	264	10.1%	46

Summary data for BMI and glycaemic associated traits obtained from genome-wide association studies (GWAS), which were utilized for Mendelian randomization. Childhood body size was categorized as either ‘thinner’, ‘about average’, or ‘plumper’, as per the GWAS data. Adult body size was determined as BMI categorized into childhood body size proportions as per the GWAS data. F statistic >10 suggests adequate instrument strength. SHBG: sex-hormone binding globulin; SNP: single-nucleotide polymorphism; %: percentage; R2: variance explained; *F*: F statistic; NA: not applicable. *Data sources*: Pulit *et al.* (2019) and Richardson *et al.* (2020).

#### Instrument identification and data harmonization

We selected independent (linkage disequilibrium (LD) threshold of *r*^2^ < 0.001 using PLINK and phase 3 version 5 of the 1000 genomes project as reference panel) genome-wide significant (*P* < 5 × 10^−8^) single-nucleotide polymorphisms (SNPs). The number of variants for each exposure is summarized in Table I. For multivariable MR, we repeated LD clumping for the combined set of SNPs from all exposures included in the model. All effect alleles were checked to be on the forward strand; ambiguous palindromes were not excluded. Where possible, SNPs absent in one of the exposure-outcome sets were proxied using variants in LD (*r*^2^ > 0.8).

### Statistical analysis

Variance explained (*r*^2^) was calculated using 2EAF(1 − EAF)β^2^, where EAF is the effect allele frequency. F statistic was derived using (*r*^2^/*K*)/[(1 − *r*^2^)(N − *K* − 1)], where *K* is the number of SNPs and N the sample size. Conditional F statistics was derived using the R MVMR package (R Foundation for Statistical Computing, Vienna, Austria) for multivariable MR. F statistics >10 is considered suggestive of adequate instrument strength. The inverse variance weighted (IVW) method was used for the primary analysis, which provides a weighted average of variant effects analogous to random-effect meta-analysis (Burgess *et al.*, 2013). Effect sizes are interpreted as per unit increase in the exposure, the definition of which is given in Table I. We used the weighted median, weighed mode and MR Egger methods to evaluate the robustness of IVW estimates to horizontal pleiotropy (Bowden *et al.*, 2015). Horizontal pleiotropy is a main source of bias in MR, whereby genetic variants influence the exposure and outcome via two separate biological pathways (Verbanck *et al.*, 2018). For MVMR, we used the IVW method, with MR-Egger as sensitivity analysis (Bowden *et al.*, 2015). The pairwise covariance between SNP associations was assumed to be zero in the primary analysis. All analyses were performed in R (R Foundation for Statistical Computing, Vienna, Austria) using the TwoSampleMR and MVMR packages.

## Results

### Characteristics of included studies

**Figure 1.**
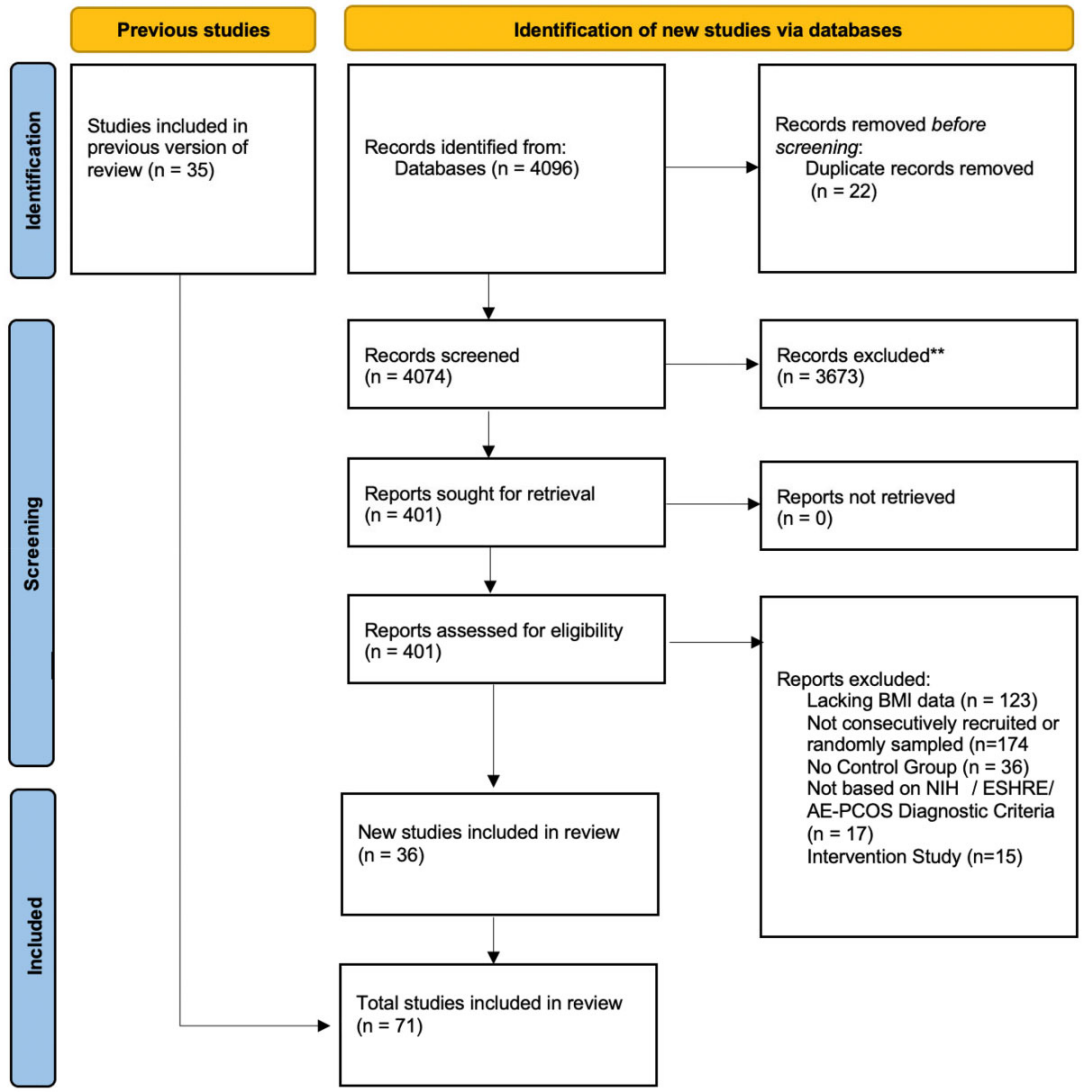
**Systematic review PRISMA flow diagram.** PRISMA flow diagram created from a template by Page *et al.* (2021). Previous studies included are from Lim *et al.* (2012). AE-PCOS: androgen excess PCOS; NIH: National Institutes of Health.

The search yielded 4074 citations from which (based on our selection criteria) 36 studies were included (Fig. 1). These studies were combined with 35 studies from the prior systematic review by Lim *et al.* (2012), providing 71 studies in total (Supplementary Tables SIV, SV, and SVI).

Thirty-five studies were included for meta-analysis of overweight (BMI ≥ 25 or ≥23 kg/m^2^ for Asian subjects) versus non-overweight in women with/without PCOS (n = 29 adult, n = 6 adolescent). Thirty studies were included for meta-analysis of obesity (BMI ≥ 30 or BMI ≥25 kg/m^2^ for Asian subjects) versus non-obese in women with/without PCOS (n = 25 adult, n = 5 adolescent). Sixteen studies were included for meta-analysis of central obesity versus non-central obesity in women with/without PCOS (n = 12 adult, n = 4 adolescent).

In terms of study design, 47.9% (34/71) were cross-sectional, 16.9% (12/71) retrospective, 22.5% (16/71) case-control, and 12.7% (9/71) prospective cohort studies. In terms of geographic distribution, 36.6% (26/71) were conducted in Europe (29.6% (21/71) Asia, 28.2% (20/71) Americas, 5.6% (4/71) Oceania) (Supplementary Tables SIV and SV). For quality assessment, 60.6% (43/71) were high-quality (≥5/9) and 39.4% (28/71) were low-quality (range 2/9 to 8/9) (Supplementary Table SVI). Funnel plots for overweight, obesity, and central obesity meta-analyses (Supplementary Figs S3, S4, and S5) reported no statistical evidence of heterogeneity. However, the Funnel plot of the overweight meta-analysis trended towards significance (*P* = 0.0624, Supplementary Fig S3).

### Meta-analyses of odds of PCOS in women of each weight category

#### Overweight

**Figure 2.**
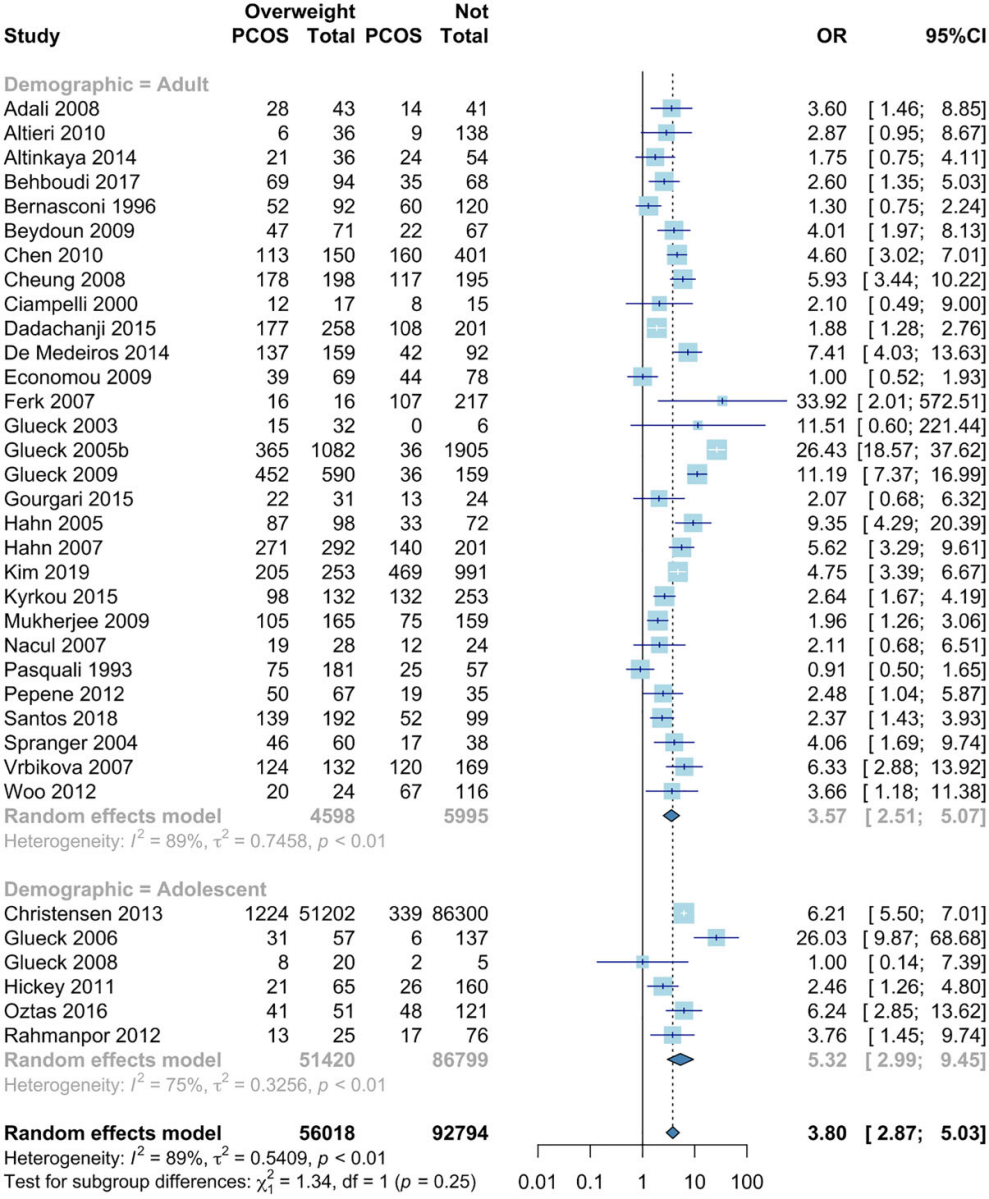
**Meta-analysis of odds of PCOS in women with overweight versus non-overweight.** Forest plot for odds ratio of PCOS in women with overweight versus non-overweight. Quantifying heterogeneity: τ^2^ = 0.5409, *I*^2^ = 89%, Test of heterogeneity: *P*-value <0.0001. OR: odds ratio.

In the 35 studies providing overweight data, odds of PCOS were significantly higher in women with overweight (OR 3.80, 95% CI: 2.87, 5.03) (Pasquali *et al.*, 1993; Bernasconi *et al.*, 1996; Ciampelli *et al.*, 2000; Glueck *et al.*, 2003, 2005b, 2006, 2008, 2009; Spranger *et al.*, 2004; Hahn *et al.*, 2005, 2007; Ferk *et al.*, 2007; Nácul *et al.*, 2007; Vrbikova *et al.*, 2007; Adali *et al.*, 2008; Cheung *et al.*, 2008; Beydoun *et al.*, 2009; Economou *et al.*, 2009; Mukherjee *et al.*, 2009; Altieri *et al.*, 2010; Chen *et al.*, 2010; Hickey *et al.*, 2011; Pepene, 2012; Rahmanpour *et al.*, 2012; Woo *et al.*, 2012; Christensen *et al.*, 2013; Altinkaya *et al.*, 2014; de Medeiros *et al.*, 2014; Dadachanji *et al.*, 2015; Gourgari *et al.*, 2015; Kyrkou *et al.*, 2016; Oztas *et al.*, 2016; Behboudi-Gandevani *et al.*, 2017; Santos *et al.*, 2018; Kim *et al.*, 2019). Odds of PCOS were higher for adolescents with overweight (adult: OR: 3.57, 95% CI 2.51, 5.07, versus adolescent: OR 5.32, 95% CI 2.99, 9.45) (lower part, Fig. 2). Five of the six childhood/adolescence studies showed that overweight increased odds of PCOS (Glueck *et al.*, 2006; Hickey *et al.*, 2011; Rahmanpour *et al.*, 2012; Christensen *et al.*, 2013; Oztas *et al.*, 2016). One study found that overweight does not increase odds of PCOS; however, this was the smallest study of this sub-group (Glueck *et al.*, 2008). Women with overweight had increased odds of PCOS across all included study types (Supplementary Fig. S6). There was significant statistical heterogeneity (*I*^2^ = 89%, *P* < 0.0001).

#### Obesity

**Figure 3.**
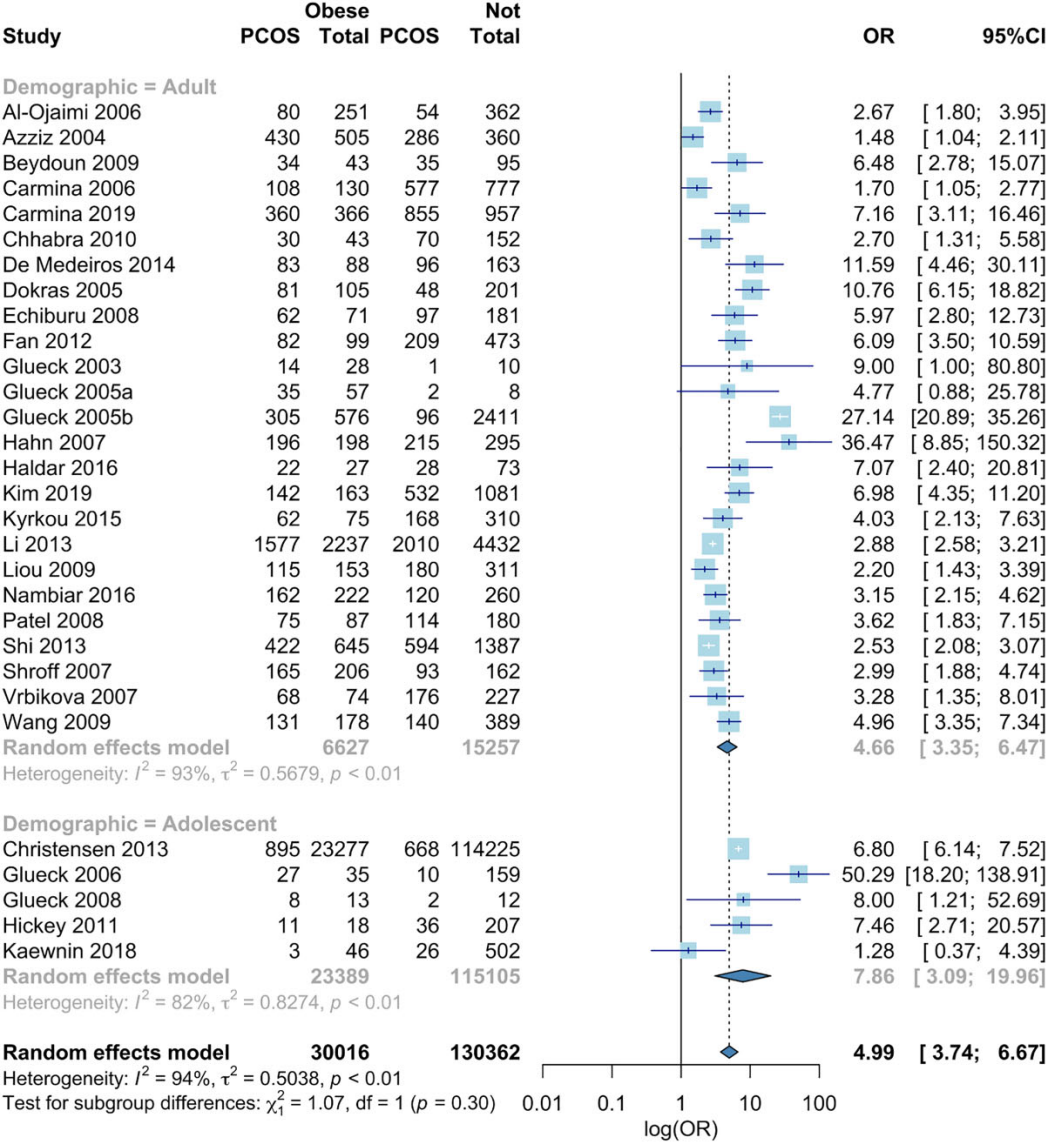
**Meta-analysis of odds of PCOS in women with obesity versus non-obesity.** Forest plot for odds ratio of PCOS in women with obesity vs non-obesity, Quantifying heterogeneity: τ^2^ = 0.5038, *I*^2^ = 94.0%, Test of heterogeneity: *P*-value <0.0001. OR: odds ratio.

In the 30 studies providing obesity data, the odds of PCOS were significantly higher in women with obesity (OR 4.99, 95% CI 3.74, 6.67) (Glueck *et al.*, 2003, 2005b, 2005a, 2006, 2009; Azziz *et al.*, 2004a; Dokras *et al.*, 2005; Al-Ojaimi, 2006; Carmina *et al.*, 2006, 2019; Hahn *et al.*, 2007; Shroff *et al.*, 2007; Vrbikova *et al.*, 2007; Echiburú *et al.*, 2008; Patel *et al.*, 2008; Beydoun *et al.*, 2009; Liou *et al.*, 2009; Wang *et al.*, 2009; Chhabra and Venkatraman, 2010; Hickey *et al.*, 2011; Fan *et al.*, 2012; Christensen *et al.*, 2013; Li *et al.*, 2013; Shi *et al.*, 2013; de Medeiros *et al.*, 2014; Kyrkou *et al.*, 2016; Nambiar *et al.*, 2016; Haldar *et al.*, 2018; Kaewnin *et al.*, 2018; Kim *et al.*, 2019) (Fig. 3). Odds of PCOS were higher in adolescents with obesity (Adult: OR 4.66, 95% CI 3.35, 6.47, Adolescent: OR 7.86, 95% CI 3.09, 19.96) (lower part, Fig. 3). Four out of five of the childhood/adolescence studies showed that obesity increased odds of PCOS (Glueck *et al.*, 2006, 2008; Hickey *et al.*, 2011; Christensen *et al.*, 2013). One study showed no association between adolescent obesity and odd of PCOS; however, the OR trended towards significance (Kaewnin *et al.*, 2018). Women with obesity had increased odds of PCOS across all included study types (Supplementary Fig. S7). There was significant heterogeneity (*I*^2^ = 94%, *P* < 0.0001).

#### Central obesity

**Figure 4.**
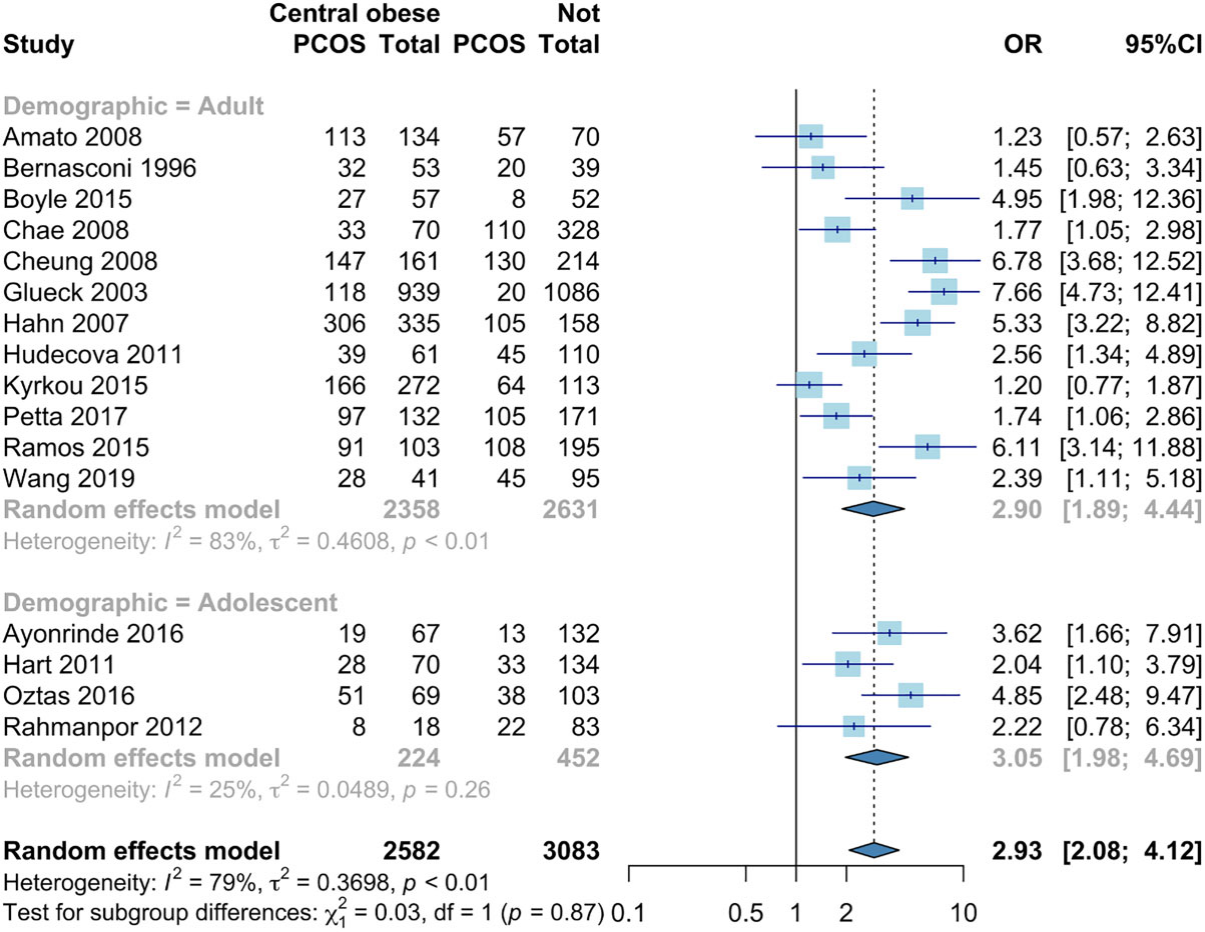
**Meta-analysis of odds of PCOS in women with central obesity versus non-central obesity.** Forest plot for odds ratio of PCOS in women with central obesity versus non-central obesity, Quantifying heterogeneity: τ^2^ = 0.3698, *I*^2^ = 79%, Test of heterogeneity: *P*-value <0.0001. OR: odds ratio.

In the 16 studies providing central obesity data, the odds of PCOS were significantly higher in women with central obesity (OR 2.93, 95% CI 2.08, 4.12) (Bernasconi *et al.*, 1996; Glueck *et al.*, 2003; Hahn *et al.*, 2007; Amato *et al.*, 2008; Chae *et al.*, 2008; Cheung *et al.*, 2008; Hart *et al.*, 2011; Hudecova *et al.*, 2011; Rahmanpour *et al.*, 2012; Boyle *et al.*, 2015; Ramos and Spritzer, 2015; Ayonrinde *et al.*, 2016; Kyrkou *et al.*, 2016; Oztas *et al.*, 2016; Petta *et al.*, 2017; Wang *et al.*, 2019) (Fig. 4). When analysed by age, the odds of PCOS were similar for both adolescents and adults (Adult: OR 2.90, 95% CI 1.89, 4.44, Adolescent: OR 3.05, 95% CI 1.98, 4.69) (lower part, Fig. 4). Three of the four childhood/adolescent studies showed that central obesity increased odds of PCOS (Hart *et al.*, 2011; Ayonrinde *et al.*, 2016; Oztas *et al.*, 2016). One study found no significant association but trended towards significance (Rahmanpour *et al.*, 2012). Women with central obesity had increased odds of PCOS across all included study types (Supplementary Fig. S8). There was significant statistical heterogeneity (*I*^2^ = 79%, *P* < 0.0001).

### Systematic review of childhood/adolescent body composition on odds of PCOS

Our review highlighted 16 studies investigating the role of childhood/adolescent body composition in PCOS risk; 9 of these were included for systematic review only and 7 were included for both systematic review and meta-analysis (Hart *et al.*, 2011; Rahmanpour *et al.*, 2012; Christensen *et al.*, 2013; Roe *et al.*, 2013; Gümüş *et al.*, 2015; Ayonrinde *et al.*, 2016; Çinar *et al.*, 2016; Oztas *et al.*, 2016; de Zegher *et al.*, 2017; Li *et al.*, 2017; Ates *et al.*, 2018; Bedaiwy *et al.*, 2018; Kaewnin *et al.*, 2018; Wu *et al.*, 2018; Koivuaho *et al.*, 2019; Aarestrup *et al.*, 2021). Of note, a large study of adolescent girls demonstrated that PCOS was preceded by a marked z-score increase between birthweight and BMI at diagnosis (de Zegher *et al.*, 2017). Similarly, in a Danish study (n = 65 665), girls age 7–13 years with overweight were at higher risk (Age 7 years: hazard ratio (HR): 2.83, Age 13 years: HR 2.99) of PCOS than those without overweight, with overweight at both time points further increasing the risk (Aarestrup *et al.*, 2021). Christensen *et al.* (2013) (n = 138 502 adolescents age 15–19) demonstrated that higher BMI category increased the PCOS risk further (OR compared to normal weight, overweight: OR 3.85, obese: OR 10.25, extreme obesity: OR 23.1). Furthermore, a Northern Finland longitudinal study demonstrated that an earlier age of fat gain and higher BMI in childhood increased PCOS risk (Koivuaho *et al.*, 2019).

### Mendelian randomization

The number of variants used to instrument each exposure, and their respective F statistics, are summarized in Table I.

#### BMI

**Figure 5.**
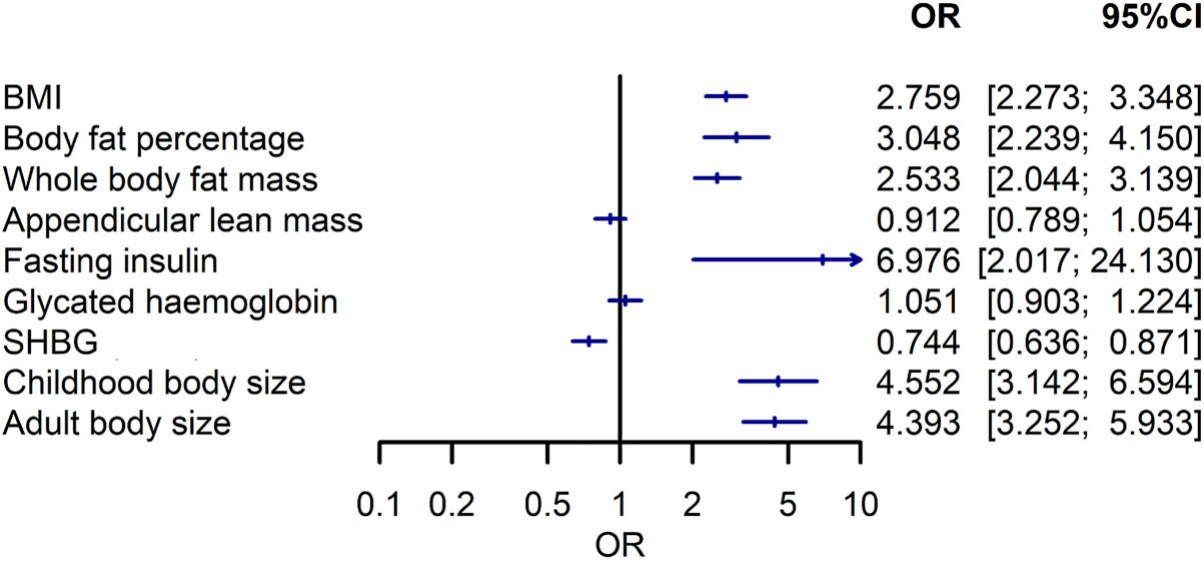
**Two sample Mendelian randomization analysis of body composition and metabolic parameters on odds of PCOS.** Forest plot of impact of exposure (body composition, metabolic parameters) on PCOS odds. SHBG: sex hormone-binding globulin; OR: odds ratio.

Each standard deviation (4.8 kg/m^2^) increase in BMI was associated with 2.8-fold higher odds of PCOS (OR: 2.76, 95% CI: 2.27–3.35) (Fig. 5).

#### Fat and fat-free mass

Each standard deviation increase in body fat percentage (OR 3.05 per 8.5% increase; 95% CI: 2.24–4.15) and whole-body fat mass (OR 2.53 per 9.6 kg increase; 95% CI: 2.04–3.14) were significantly associated with odds of PCOS. By contrast, appendicular lean mass was not associated with PCOS (OR 0.91 per 5.6 kg increase; 95% CI: 0.79–1.05) (Fig. 5).

#### Childhood and adult body size

Each increase in category of genetically predicted childhood (OR: 4.55, 95% CI: 3.14–6.59) and adult body sizes (OR: 4.39, 95% CI: 3.25–5.93) were associated with odds of PCOS in univariable MR models (Fig. 5). In MVMR, conditional F statistics were 11 and 13 for childhood and adult body sizes, respectively, suggesting adequate instrument strength. Childhood body size had an independent effect on PCOS after accounting for adult body size (OR 2.56; 95% CI: 1.57, 4.20).

#### Effect of SHBG and fasting insulin on PCOS

Fasting insulin (OR: 6.98 per 0.79 pmol/l increase; 95% CI: 2.02–24.13) and SHBG levels (OR: 0.74 per 28 nmol/l increase, 95% CI: 0.64–0.87) were associated with odds of PCOS. Glycated haemoglobin was not associated with PCOS odds (OR: 1.05 per 6.7 mmol/mol increase, 95% CI: 0.90–1.22) (Fig. 5).

## Discussion

Using two complementary methodologies (systematic review/meta-analysis and MR), we provide robust evidence in support of a strong causal association between first, overweight/obesity, and specifically central fat accumulation, and second, metabolic parameters suggestive of insulin resistance (including hyperinsulinaemia and low SHBG), with incident PCOS. Importantly, our study demonstrates for the first time a critical role for the impact of excess childhood/adolescent adiposity in the pathophysiology of adult PCOS. This novel finding starkly reminds us of the adult implications of living with overweight and obesity in childhood and adolescence, besides its effects in adulthood, on the risk of incident PCOS.

Studies selected via systematic review suggest an association between abnormal childhood/adolescent body composition and odds of PCOS. A two-cohort study from the systematic review demonstrated an increased risk of PCOS when there was a marked Z score increase in birthweight (de Zegher *et al.*, 2017). Additionally, Christensen *et al.*’s cross-sectional study (n = 138 502) reported that a longer duration of living with overweight increased PCOS risk in a linear relation (Christensen *et al.*, 2013). In fact, adolescents with severe obesity (BMI ≥ 40 kg/m^2^) had a 23.1-fold increased odds of PCOS (overweight OR 3.85, obese OR 10.25). Notably, the study design limits determination of causality between exposure (BMI) and outcome (Christensen *et al.*, 2013). Our MR data implicates early life body size in PCOS risk, independent of adult body size. Importantly, this suggests that young females with overweight and obesity, even following reversion to a normal adult weight, still have an increased PCOS risk. Overall, there is a clear causal relation between aspects of abnormal body composition during childhood, adolescence and adulthood, and incident PCOS.

In general, BMI increases rapidly during the first year of life, then subsequently decreases and reaches a nadir at ∼6 years. Thereafter, BMI increases again throughout childhood, and this second rise is referred to as the adiposity rebound. An earlier adiposity rebound is implicated as a predictive marker of obesity in later childhood, adolescence, and adulthood (Kang, 2018). Age at adiposity rebound is also associated with PCOS diagnosis: in the Northern Finland cohort study of 280 women with PCOS and 1573 controls, women with PCOS had lower birthweight, earlier adiposity rebound and higher subsequent BMI versus control. One proposed underlying mechanism linking low-birthweight individuals with subsequent metabolic disease relates to a reduced capacity for subcutaneous fat storage. This means during subsequent weight gain and accretion of fat mass, especially occurring at an early age, fat is partitioned to the ectopic sites, including the liver and visceral adipose tissue, driving systemic insulin resistance and contributing to PCOS development (Koivuaho *et al.*, 2019). This mechanism in children of low birthweight suggests that targeting metabolic abnormalities and weight maintenance is particularly important in this subgroup. This has been shown with metformin therapy in low-birthweight girls, and with weight loss in adults/adolescents with PCOS and obesity, with reduced adiposity and insulin sensitizing addressing hyperandrogenism, insulin resistance and restoring normal ovulatory function (Gambineri *et al.*, 2002; Kuchenbecker *et al.*, 2011; Lass *et al.*, 2011). The contribution of lifestyle factors, diet, physical activity, and sedentary behaviour to BMI in women with and without PCOS was convincingly shown in the Australian Longitudinal Study of Women’s Health (Moran *et al.*, 2013).

SHBG is an important surrogate marker of insulin resistance, which mediates the hyperandrogenism in PCOS. Our MR data reports that lower genetically determined SHBG increases PCOS odds. Similarly, a MR study reported that a 1 standard deviation higher genetically determined testosterone increased PCOS odds (OR: 1.51, 1.33–1.72) (Ruth *et al.*, 2020). Two cross-sectional studies also associate peripubertal obesity with hyperandrogenaemia and hyperinsulinaemia in early puberty; this supports excess adiposity as predisposing to hyperandrogenism and metabolic dysfunction (Laitinen *et al.*, 2003; McCartney *et al.*, 2006, 2007). Furthermore, in the Northern Finland birth cohort, weight gain and hyperandrogenism during early adulthood (age 14–31 years) were associated with PCOS diagnosis and symptoms (Ollila *et al.*, 2016). These data add to the strength of evidence suggesting adolescent hyperandrogenism is a biochemical precursor of PCOS, occurring with weight gain. This is important given the exponentially increasing obesity epidemic, particularly in younger people.

Phenotypic clustering analysis has afforded new mechanistic insights into the pathophysiology of PCOS and the existence of two potential PCOS phenotypes: metabolic and reproductive phenotypes (Dapas *et al.*, 2020). While the reproductive phenotype is characterized by lesser adiposity, a relatively low BMI, normal insulin levels and increased LH and SHBG concentrations, the metabolic phenotype is characterized by higher BMI and hyperinsulinaemia but low LH and SHBG (Dapas *et al.*, 2020; Yan *et al.*, 2021). Our results, driven by genetically determined childhood/adolescent body composition, higher BMI, hyperinsulinaemia, and lower SHBG, clearly favour obesity driving the metabolic, but not reproductive, PCOS phenotype. Further research should investigate the observational and genetic epidemiology of the potential PCOS phenotypes.

### Strengths and limitations

There are several strengths of this study. First, we combine two different methodological approaches, namely MR and meta-analysis, facilitating determination of the mediating effect of excess adiposity on PCOS risk. Second, our systematic review screening criteria were based on a previously published high-quality systematic review/meta-analysis, updating it *c.*10 years later. Finally, we used a two-sample MR approach, which is more robust against confounding and reverse causation compared to traditional observational designs.

The study has several limitations. First, the optimal MA would have been of prospective studies only. Given that these studies were limited, we also included cross-sectional and retrospective case-control studies and, as such, this limits our ability to determine causality between exposure (body composition) and outcome (PCOS). However, sensitivity analysis demonstrated the meta-analysis findings persisted across all study types (including prospective cohort studies). Second, ideally all patients would have been recruited from the general population to minimize the selection biases. In our study, a large proportion of studies recruited patients from hospital clinics and specific demographics. There is a risk that the recruited population does not reflect the population’s overall exposure rates. Third, there was a smaller number of studies of adolescents than adults, such that for adolescents the MA measure of PCOS odds is less precise (with wider CIs). In terms of MR, the analyses were based on PCOS classified by both diagnostic criteria and self-diagnosis, which may have potentially biased findings, including individuals not fulfilling the diagnostic criteria utilized in the systematic review. Furthermore, the current analysis estimates the effect of various exposure on PCOS susceptibility, which may not generalize to PCOS progression. Adiposity is likely to have a non-linear association with PCOS, which should be the focus of future studies. MR was analysed on a continuous scale (per unit increase in standard deviation), whereas meta-analysis was analysed using categorical data. This may limit the integration of results. The study population included mainly participants of European ancestry; future study among other ethnic populations may be helpful in confirming the generalizability of the current findings. Finally, GWAS of BMI did not exclude PCOS from the analyses. However, PCOS prevalence would be low in the total cohort, which includes males and females, and there is greater causality for BMI causing PCOS given the observational data Therefore, risk of bias due to not excluding individuals with PCOS from analyses is unlikely.

## Conclusion

In conclusion, from several sources of evidence, childhood, adolescent, and adulthood overweight and obesity drive a distinct metabolic phenotype of PCOS, with implications for early weight maintenance to minimize the risk of subsequently developing PCOS and later cardiometabolic complications.

## Supplementary Material

dead053_Supplementary_Figure_S1

dead053_Supplementary_Figure_S2

dead053_Supplementary_Figure_S3

dead053_Supplementary_Figure_S4

dead053_Supplementary_Figure_S5

dead053_Supplementary_Figure_S6

dead053_Supplementary_Figure_S7

dead053_Supplementary_Figure_S8

dead053_Supplementary_Table_SI

dead053_Supplementary_Table_SII

dead053_Supplementary_Table_SIII

dead053_Supplementary_Table_SIV

dead053_Supplementary_Table_SV

dead053_Supplementary_Table_SVI

## Data Availability

Data for the systematic review and Mendelian randomization are available upon reasonable request to the corresponding author.
