# Supplementary material for: Childhood, adolescent, and adulthood adiposity are associated with risk of PCOS: a Mendelian randomization study with meta-analysis
Source: Hum Reprod. 2023 Apr 4;38(6):1168–82. doi: 10.1093/humrep/dead053 (PMC10233304; doi:10.1093/humrep/dead053)
Supplement: dead053_Supplementary_Figure_S1 [file dead053_supplementary_figure_s1.pdf]

Human Reproduction, pp. 1–1, 2023
https://doi.org/10.1093/humrep/dead053

human reproduction

SUPPLEMENTARY DATA

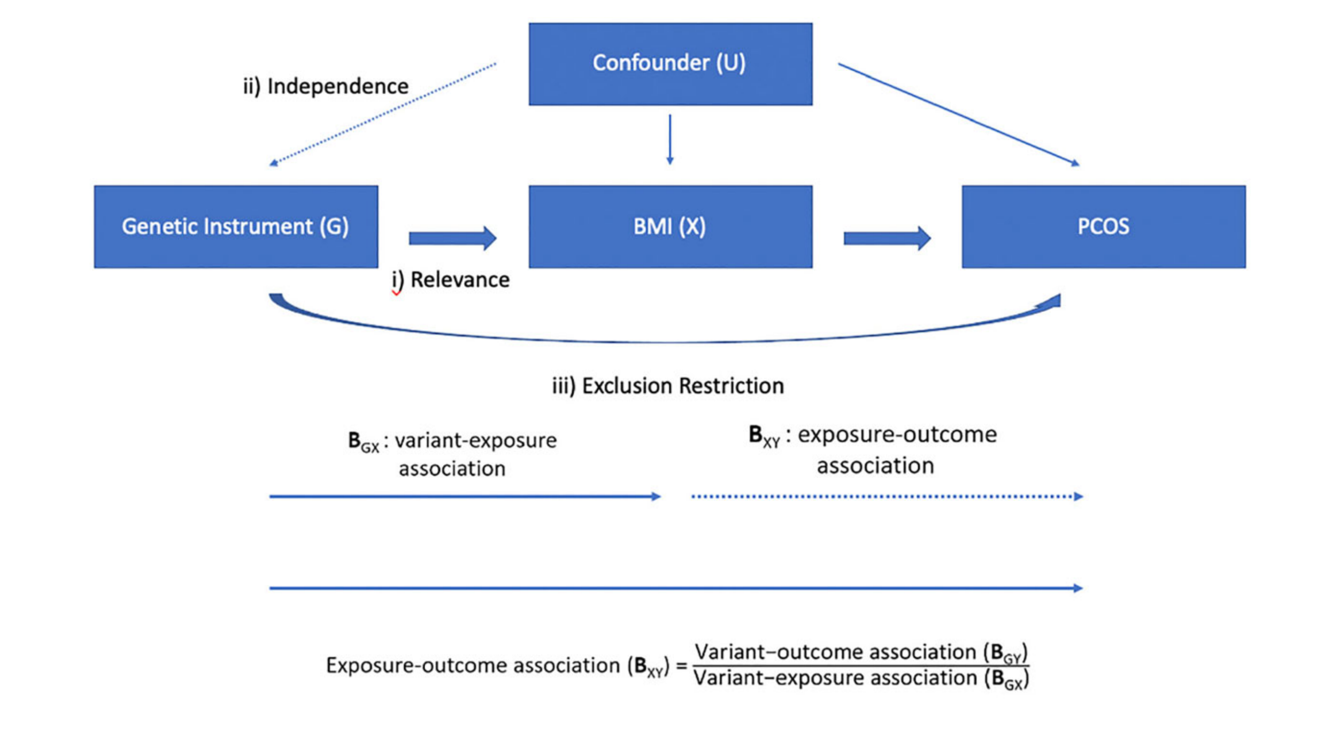

Supplementary Figure S1. Explanation of Mendelian randomization technique. Mendelian randomization (MR) is an observational design that is less likely to be biased by confounding or reverse causation than conventional observational studies because the genetic variants are randomly allocated at conception with respect to environmental confounders. The exposure–outcome (e.g. BMI-PCOS) association, B_{XY}, can be derived using the ratio method. As summarized in Supplementary Fig. S1, B_{XY} can be estimated by a ratio of the instrument-outcome and instrument-exposure associations. $B_{GY} = B_{GX} * B_{XY}$, therefore $B_{XY} = B_{GY}/B_{GX}$. A genetic variant can be considered as a valid instrumental variable for an exposure if it satisfies the instrumental variable assumptions: it is associated with the exposure (relevance) in a specific way that does not affect the outcome except via the exposure (exclusion-restriction), and it is not associated with the outcome due to confounding (independence).
