# Supplementary material for: Childhood, adolescent, and adulthood adiposity are associated with risk of PCOS: a Mendelian randomization study with meta-analysis
Source: Hum Reprod. 2023 Apr 4;38(6):1168–82. doi: 10.1093/humrep/dead053 (PMC10233304; doi:10.1093/humrep/dead053)
Supplement: dead053_Supplementary_Figure_S2 [file dead053_supplementary_figure_s2.pdf]

Human Reproduction, pp. 1–1, 2023
https://doi.org/10.1093/humrep/dead053

human reproduction

SUPPLEMENTARY DATA

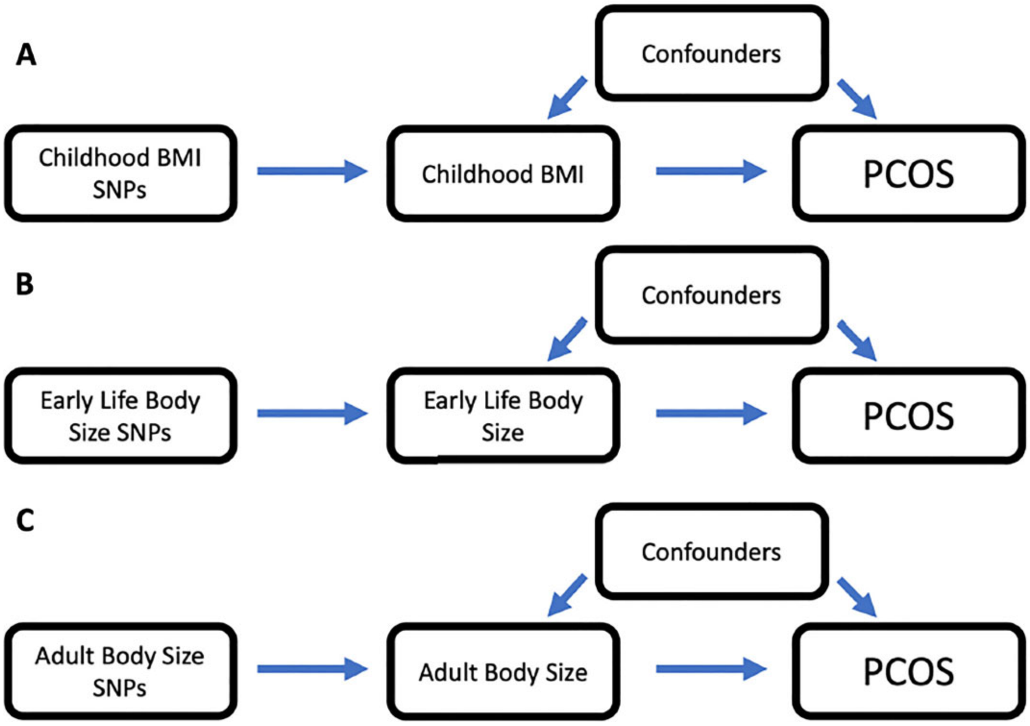

Supplementary Figure S2. Schematic representation of MR analyses. PCOS: polycystic ovary syndrome; SNP: single-nucleotide polymorphism; MR: Mendelian randomization.
