# Supplementary material for: Childhood, adolescent, and adulthood adiposity are associated with risk of PCOS: a Mendelian randomization study with meta-analysis
Source: Hum Reprod. 2023 Apr 4;38(6):1168–82. doi: 10.1093/humrep/dead053 (PMC10233304; doi:10.1093/humrep/dead053)
Supplement: dead053_Supplementary_Figure_S4 [file dead053_supplementary_figure_s4.pdf]

Human Reproduction, pp. 1–1, 2023
https://doi.org/10.1093/humrep/dead053

human
reproduction

SUPPLEMENTARY DATA

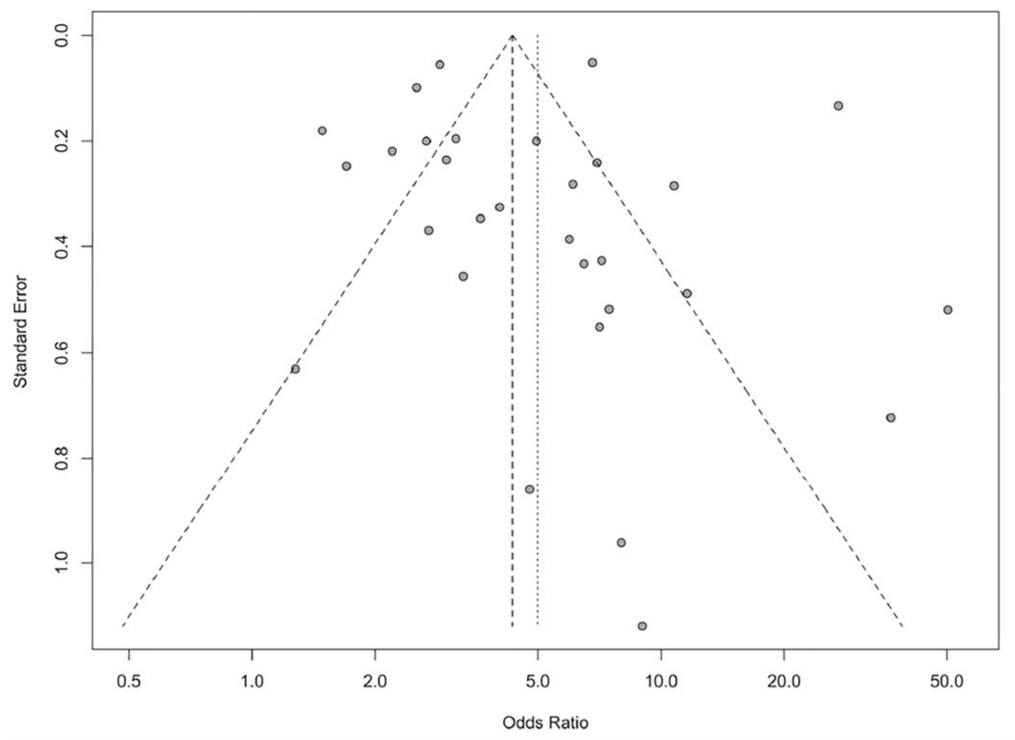

Supplementary Figure S4. Funnel plot of obesity meta-analysis. *X*-axis = non-linear scale. Thin dotted line = mean odds ratio. Egger regression test of funnel plot asymmetry. Test result: $t = 0.31$, $df = 28$, P-value = 0.7558.
