# Supplementary material for: Childhood, adolescent, and adulthood adiposity are associated with risk of PCOS: a Mendelian randomization study with meta-analysis
Source: Hum Reprod. 2023 Apr 4;38(6):1168–82. doi: 10.1093/humrep/dead053 (PMC10233304; doi:10.1093/humrep/dead053)
Supplement: dead053_Supplementary_Figure_S8 [file dead053_supplementary_figure_s8.pdf]

Human Reproduction, pp. 1–1, 2023
https://doi.org/10.1093/humrep/dead053

human
reproduction

SUPPLEMENTARY DATA

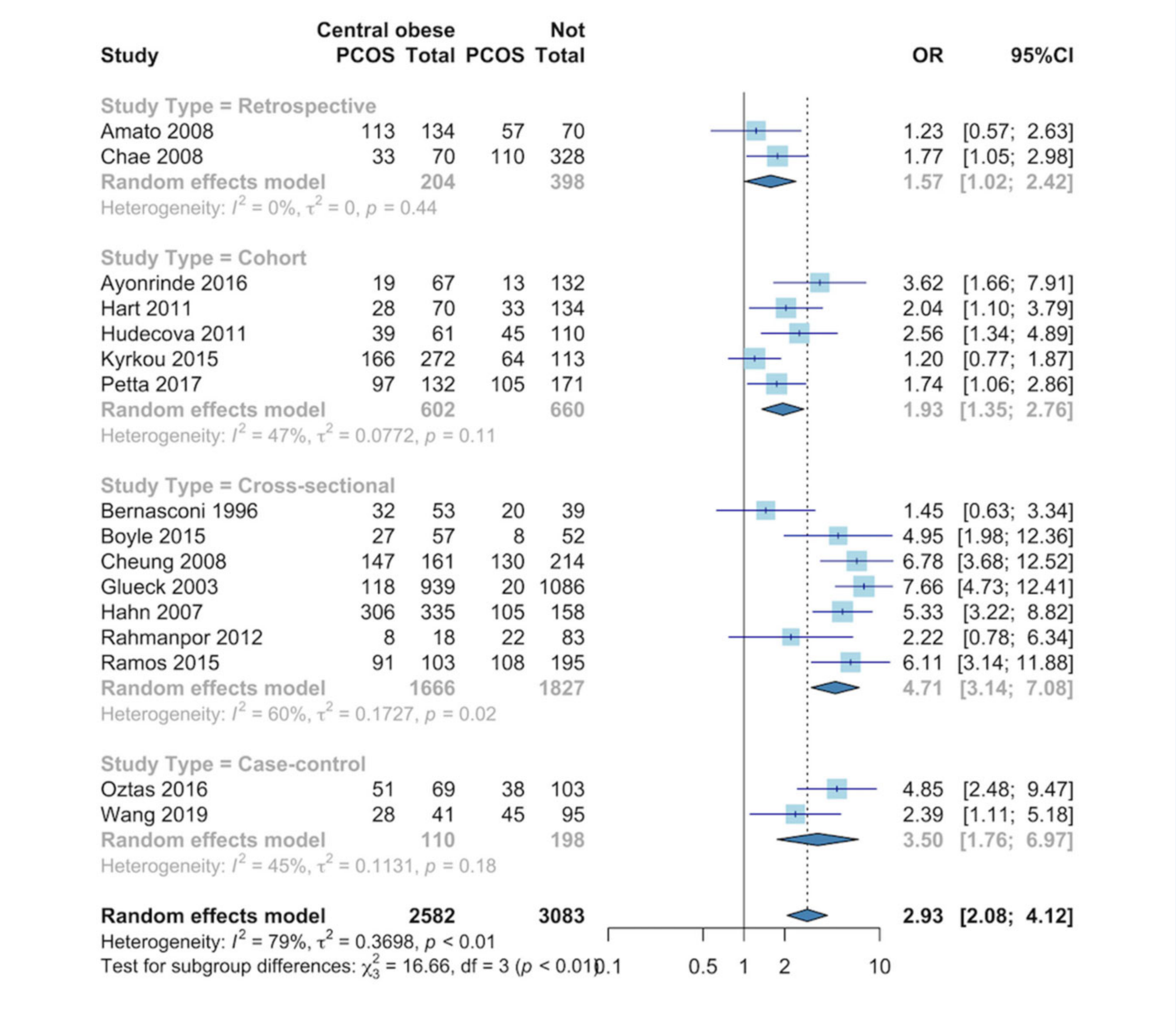

Supplementary Figure S8. Central obesity meta-analysis by study type. Forest plot for odds ratio of PCOS in central obese vs non-central obese. OR: odds ratio.
