# Supplementary material for: Childhood, adolescent, and adulthood adiposity are associated with risk of PCOS: a Mendelian randomization study with meta-analysis
Source: Hum Reprod. 2023 Apr 4;38(6):1168–82. doi: 10.1093/humrep/dead053 (PMC10233304; doi:10.1093/humrep/dead053)
Supplement: dead053_Supplementary_Table_SI [file dead053_supplementary_table_si.pdf]

Human Reproduction, pp. 1–1, 2023
https://doi.org/10.1093/humrep/dead053

human reproduction

SUPPLEMENTARY DATA

Supplementary Table SI MeSH terms.

1	exp Polycystic Ovary Syndrome/
2	PCO$.tw
3	polycystic ovar$.tw
4	(stein-leventhal or leventhal).tw
5	(ovar$ adj3 (sclerocystic or polycystic or degeneration)).tw
6	or/1-5
7	exp Obesity/
8	exp Overweight/
9	exp Body Mass Index/
10	exp Body Weight/
11	(obes$ or overweight$ or adipo$).tw
12	over weight.tw
13	((increas$ or excess$) adj3 weight).tw
14	((increas$ or excess$ or high) adj3 BMI).tw
15	((increas$ or excess$ or high) adj3 (body mass index)).tw
16	exp Adipose tissue/
17	exp Abdominal fat/
18	exp Skinfold Thickness/
19	exp Waist-Hip Ratio/
20	waist-hip ratio.tw
21	WHR.tw
22	skinfold.tw
23	abdom$.tw
24	waist.tw
25	fat.tw
26	DEXA.tw
27	anthropometry.tw
28	bioimpedance.tw
29	or/7-28
30	6 and 29
31	animal/
32	human/
33	31 not (31 and 32)
34	30 not 33

MeSH: Medical Subject Heading for Medline; Exp: exploded MeSH; tw: text word; adj: adjacency; $: any character (taken from supplementary material by Lim *et al.* (2012, *Human Reproduction Update*).
