# Supplementary material for: Childhood, adolescent, and adulthood adiposity are associated with risk of PCOS: a Mendelian randomization study with meta-analysis
Source: Hum Reprod. 2023 Apr 4;38(6):1168–82. doi: 10.1093/humrep/dead053 (PMC10233304; doi:10.1093/humrep/dead053)
Supplement: dead053_Supplementary_Table_SII [file dead053_supplementary_table_sii.pdf]

Human Reproduction, pp. 1–1, 2023
https://doi.org/10.1093/humrep/dead053

human reproduction

SUPPLEMENTARY DATA

Supplementary Table SII Diagnostic criteria used to classify PCOS.

Diagnostic criteria	NIH Consensus 1990 (all criteria required for diagnosis)	ESHRE/ASRM 2003 (2/3 diagnostic criteria required) (also termed Rotterdam Criteria)	AE-PCOS (hyperandrogenism + one other criteria)
Hyperandrogenism	Clinical and/or biochemical hyperandrogenism	Clinical and/or biochemical hyperandrogenism	Clinical and/or biochemical hyperandrogenism
Ovulation	Oligo/amenorrhoea, anovulation	Oligo/amenorrhoea, anovulation	Oligo/amenorrhoea, anovulation
Pelvic ultrasound		Polycystic ovaries on ultrasound	Polycystic ovaries on ultrasound
Exclusion of other endocrinopathies for diagnosis?	Yes	Yes	Yes

PCOS: polycystic ovary syndrome; NIH: National Institute for Health; ESHRE/ASRM: European Society of Human Reproduction and Embryology/American Society of Reproductive Medicine Criteria; AE-PCOS: Androgen Excess PCOS.
Adapted from Spritzer (2014).
