# Supplementary material for: Childhood, adolescent, and adulthood adiposity are associated with risk of PCOS: a Mendelian randomization study with meta-analysis
Source: Hum Reprod. 2023 Apr 4;38(6):1168–82. doi: 10.1093/humrep/dead053 (PMC10233304; doi:10.1093/humrep/dead053)
Supplement: dead053_Supplementary_Table_SIII [file dead053_supplementary_table_siii.pdf]

Human Reproduction, pp. 1–1, 2023
https://doi.org/10.1093/humrep/dead053

human reproduction

SUPPLEMENTARY DATA

Supplementary Table SIII **Body composition definitions.**

Variable	Sub-group	Definition
**Overweight**	Adults (of all ethnicities other than Asian ethnicity)	BMI $\geq$ 25 kg/m^2 (World Health Organization Criteria)
	Adults of Asian ethnicity	BMI $\geq$ 23 kg/m^2 (International Obesity Task Force)
	Adolescents	85–95th percentile of age-gender-specific percentile BMI distributions
**Obesity**	Adults (of all ethnicities other than Asian ethnicity)	BMI $\geq$ 30 kg/m^2 (World Health Organization Criteria)
	Adults of Asian ethnicity	BMI $\geq$ 25 kg/m^2 (International Obesity Task Force)
	Adolescents	$\geq$ 95 percentile of age-gender specific percentile BMI distributions
**Central Obesity**	Multiple definitions utilized according to data presented in the Study	a. WC >88 cm (Adult Treatment Panel III)<br>b. WHR >0.85<br>c. WC $\geq$80 cm (International Diabetes Federation)

WC: waist-circumference; WHR: waist–hip ratio.
