# Supplementary material for: Childhood, adolescent, and adulthood adiposity are associated with risk of PCOS: a Mendelian randomization study with meta-analysis
Source: Hum Reprod. 2023 Apr 4;38(6):1168–82. doi: 10.1093/humrep/dead053 (PMC10233304; doi:10.1093/humrep/dead053)
Supplement: dead053_Supplementary_Table_SIV [file dead053_supplementary_table_siv.pdf]

Human Reproduction, pp. 1–9, 2023
https://doi.org/10.1093/humrep/dead053

Supplementary Table SIV **Systematic review studies summary table.**

Author (year) DOI	PCOS (n)	Control (n)	Age, mean ± SD	PCOS definition	PCOS ethnicity	Study design; period	Country; PCOS recruitment source	Control recruitment source	Control description
**Studies from Updated Systematic Review**									
Aarestrup et al. (2021) DOI: 10.1159/000515294	503	54 797	Not stated	NIH		Cohort; January 1977–June 2017	Denmark; discharge diagnoses obtained by linked to the Danish National Patient Registry	Copenhagen School Health Records Registry	All children who attended a public or private school in Copenhagen
Altinkaya et al. (2014) DOI: 10.1016/j.ejogrb.2014.02.022	45	45	23.5 ± 5.3, 25.1 ± 5.7	ASHRE/ASRM	Not stated	Cross-sectional; not stated	Turkey; not stated	Not stated	Age-matched women who had regular menses and no clinical or biochemical hyperandrogenism or PCO
Ates et al. (2018) DOI: 10.1080/01443615.2017.1345875	77	33	17.68 ± 1.19, 17.94 ± 1.05	NIH	Not stated	Cohort; November 2012–October 2015	Turkey; Department of Gynaecology and Obstetrics at University	Same group as PCOS	Age-matched adolescent girls with regular menstrual cycles, normal ovaries on ultrasonography and the absence of symptoms of hyperandrogenism who were admitted to the clinic for other gynaecologic disorders
Ayonrinde et al. (2016) DOI: 10.1111/jgh.13241	32	167	15.3 ± 0.3	NIH	Not stated	Cohort; not stated	Australia; Western Australian Pregnancy Cohort	Same group as PCOS	
Behboudi-Gandevani et al. (2017) DOI: 10.1007/s40618-017-0650-x	104	58	29.3 ± 5.3, 31.8 ± 5.7	NIH	Not stated	Case–control; not stated	Iran; Outpatient Endocrinology Unit	Not stated	Healthy eumenorrhoeic non-hirsute women aged 20–40 years
Boyle et al. (2015) DOI: 10.1111/imj.12910	35	74	Median: 32 (IQR 21.0, 37.0), 33 (IQR: 23, 39)	NIH	Indigenous Australians	Cross-sectional; 2003–2005	Australia; Darwin Region Urban Indigenous Diabetes study participants—living in defined geographical region within Darwin area, Australia, for ≥6 months	Same group as PCOS	Non-hyperandrogenic or hyperandogenic or idiopathic hirsutism
Carmina et al. (2019) DOI: 10.1111/cen.14063	1215	108	24 ± 5, range 18–40	NIH	Not stated	Retrospective; July 2008–June 2018	Italy; Department of Health Sciences at University	Same group as control	Normal ovulatory women were selected as controls. These women were drawn from the same population base, had no complaints related to androgen excess and were matched for age with our patient population of women with PCOS
Christensen et al. (2013) DOI: 10.1016/j.fertnstert.2013.04.001	1563	135 939	17.7 ± 1.4, 17.0 ± 1.5	NIH	21.8% non-Hispanic White, 9.3% Black, 51.8% Hispanic White, 5.4% Asian/Pacific Islander, 2.3% other/multiple Races, 9.4% unknown	Cross-sectional; January 2007–December 2009	USA; subset of patients enrolled in large population-based cohort study, KPSC Children's Health Study 2007–2009. This actively enrolled children in a large, prepaid integrated managed healthcare system	Same group as PCOS	No indication of NIH criteria

(continued)

Supplementary Table SIV Continued

Author (year) DOI	PCOS (n)	Control (n)	Age, mean ± SD	PCOS definition	PCOS ethnicity	Study design; period	Country; PCOS recruitment source	Control recruitment source	Control description
Çınar *et al.* (2016) DOI: 10.1016/ j.jpag.2015.12.005	89	84	18.31 ± 0.21, 18.81 ± 0.21	ASHRE/ASRM	Not stated	Case–control; January 2014–December 2015	Turkey; outpatient adolescence clinic in tertiary referral hospital	Not stated	Normal menstrual cycles, with no evidence of hyperandrogenism and normal ovarian morphology on pelvic ultrasonography with a Ferriman–Gallwey score (FGS) < 8
Dadachanji *et al.* (2015) DOI: 10.1016/ j.fertnstert. 2015.03.037	285	174	25.93 ± 6.15, 24.97 ± 4.98	ESHRE/ASRM	Not stated	Case–control; not stated	India; Infertility and Endocrinology outpatient clinic	General population	Age-matched regularly menstruating women with absence of any clinical and/or biochemical signs of hyperandrogenaemia and having normal ovaries on ultrasound imaging, with the same ethnic background
De Medeiros *et al.* (2014) DOI: 10.1055/s-0034-1377043	179	72	27.08 ± 5.45, 30.50 ± 5.10	NIH, AE-PCOS	Not stated	Cross-sectional; January 2003–June 2012	Brazil; outpatient reproductive and endocrine clinic in a University Hospital	Same clinic as PCOS	Tubal or male cause of infertility and lack of PCOS
de Zegher *et al.* (2017) DOI: 10.1002/ oby.21935	467	87	15 ± 2, 16 ± 2, 15 ± 1	NIH	>90% Caucasian	Cross-sectional; not stated	Spain + Germany; University Hospitals in Barcelona, Spain and Datteln, Germany	Recruited from nearby schools and from hospital staff	Healthy, regular menses and had an ethnic background similar to those of PCOS
Fan *et al.* (2012) DOI: 10.1016/ j.rbmo. 2011.10.010	291	281	25.03 ± 4.04, 28.37 ± 3.92	ESHRE/ASRM	Chinese	Cross-sectional; 2006–2010	China; outpatient reproductive endocrinology department at University Hospital	Same clinic as PCOS	Infertile women due to salpingian obstruction or the husband's infertility. All were clinically healthy women who had regular menstrual cycles (<35 days), exhibited normal circulating androgen concentrations and the absence of obvious acne or hirsutism on physical examination or polycystic ovaries as determined by ultrasound
Gümüş *et al.* (2015) DOI: 10.1016/ j.jpag.2014.08.007	49	38	18.8 ± 2.2, 19.61 ± 2.41	ESHRE/ASRM	Not stated	Case-control; June 2013–December 2013	Turkey; outpatient adolescence department	Same clinic as PCOS	Patients without PCOS who had been admitted for vaginal and urinary system infections
Gourgari *et al.* (2015) DOI: 10.1210/jc.2015-2566	35	20	20.4 ± 4.0, 23.6 ± 2.6	AE-PCOS	Not stated	Cross-sectional; not stated	USA; Paediatric Endocrine Division of Infants and Children's Hospital and also from other physicians	Database at Healthy Volunteer Office of the National Institutes of Health + local advertisement.	18–29 years old with normal menstrual function and without evidence of PCOS were eligible; they had to be off oral contraceptive pills or any other medications that alter steroidogenesis for at least 1 month prior to participating
Haldar *et al.* (2018) DOI: 10.1007/s00394-016-1357-z	50	50	23.56 ± 0.68, 24.36 ± 0.78	ESHRE/ASRM	Indian	Case–control; January 2012–December 2012	India; Outpatient Department of Obstetrics and Gynaecology	Same clinic as PCOS	Age-matched healthy female volunteers with regular menstrual cycles (26–34 days), normal ovarian morphology, and normal total testosterone levels

(continued)

Supplementary Table SIV Continued

Author (year) DOI	PCOS (n)	Control (n)	Age, mean ± SD	PCOS definition	PCOS ethnicity	Study design; period	Country; PCOS recruitment source	Control recruitment source	Control description
Hart *et al.* (2011) DOI: 10.1016/j.fertnstert.2011.03.001	61	143	Age range 14–17 years	ESHRE/ASRM	Not stated	Cohort; not stated	Australia; Western Australian Pregnancy Cohort (Raine) Study	Same cohort as PCOS	
Hickey *et al.* (2011) DOI: 10.1093/humrep/der102	47	178	15.2	ESHRE/ASRM	91% Caucasian	Cohort; not stated	Australia; Western Australian Pregnancy Cohort (Raine) Study	Same cohort as PCOS	
Hudecova *et al.* (2011) DOI: 10.1016/j.fertnstert.2011.08.006	84	87	43 ± 5.8, 43.7 ± 6.2	ESHRE/ASRM	Not stated	Cohort; not stated	Sweden; out-patient register at University Hospital	Population registers	Residing in same county and born during the same month as the index patients. Healthy control status was assured by the absence of polycystic ovaries on transvaginal ultrasound. Furthermore, all control subjects denied a prior history of oligomenorrhoea or amenorrhoea (lasting more than 3 months)
Kaewnin *et al.* (2018) DOI: 10.1080/09513590.2017.1409716	29	519	18.66 ± 0.49, 18.69 ± 0.47	ESHRE/ASRM	Thai	Cross-sectional; July 2016–January 2017	Thailand; University students in Bangkok	Same group as PCOS	
Kim *et al.* (2019) DOI: 10.1016/j.fertnstert.2019.06.035	674	570	PCOS non-IR: 25.0 ± 5.8, PCOS-IR: 24.0 ± 6.6, Control: 29.0 ± 4.5	ESHRE/ASRM	Korean	Case-control; not stated	Korea; PCOS speciality clinic at University Hospital	Healthcare centre in the same University Hospital	No specific age criteria. They had regular (21–35 days) menstrual cycles, a modified Ferriman-Gallwey score < 6, and no polycystic ovary morphology on pelvic ultrasound examination. They were taking no medications and did not have physician-diagnosed diabetes
Koivuaho *et al.* (2019) DOI: 10.1038/s41366-019-0318-z	280	1573	Women with PCOS diagnosis by age 46	NIH	Finnish	Cohort; not stated	Finland; Northern Finland Birth Cohort 1966	Same cohort as PCOS	Women without any PCOS symptoms at age 31 and without PCOS diagnosis by age 46
Kyrkou *et al.* (2016) DOI: 10.1007/s00404-015-3964-y	230	155	24.1 ± 6.1, 24.7 ± 5.7	ESHRE/ASRM	Greek	Cohort; not stated	Greece; outpatient clinic at Department of Gynaecology and Obstetrics	Healthcare professionals	Healthy volunteer females (students, medical and paramedical students, nurses and doctors), who had normal ovulatory cycles (26–35 days) and no sign of hyperandrogenism
Li *et al.* (2013) DOI: 10.1371/journal.pone.0066250	3587	3082	28.35 ± 3.75, 31.33 ± 4.69	ESHRE/ASRM	Han Chinese	Cross-sectional; June 2009–May 2012	China; outpatient reproductive clinic	Not stated	Regular menstrual cycle (26–35 days) and normal ovarian morphology. Total testosterone and modified Ferriman-Gallwey score were evaluated for exclusion of hyperandrogenism.
Nambiar *et al.* (2016) DOI: 10.1016/j.ejogrb.2016.02.031	282	200	28.64 ± 5.06, 31.11 ± 5.13	ESHRE/ASRM	South Indian	Case-control; December 2010–October 2013	India; Women's health and fertility outpatient clinic	Same clinic as PCOS	Not stated

(continued)

Supplementary Table SIV Continued

Author (year) DOI	PCOS (n)	Control (n)	Age, mean ± SD	PCOS definition	PCOS ethnicity	Study design; period	Country; PCOS recruitment source	Control recruitment source	Control description
Oztas *et al.* (2016) DOI: 10.1016/ j.ejogrb.2016.01.009	89	83	18.4 ± 0.8, 18.8 ± 0.6	ESHRE/ASRM	Not stated	Case–control; April–August 2014	Turkey; outpatient reproductive endocrinology clinic	Not stated	Healthy age-matched adolescents were also recruited as control group. All adolescents were further evaluated by an experienced, single paediatrician in order to exclude any possible concomitant endocrinological and other systemic diseases. All
Pepene (2012) DOI: 10.1111/j.1365-2265.2011.04171.x	69	33	Lean PCOS: 22.74 ± 1.09, overweight PCOS: 26.66 ± 1.02, lean control: 24.63 ± 1.72, overweight control: 29.88 ± 1.95	AE-PCOS	Not stated	Case–control; not stated	Romania; not stated	Not stated	Controls had no evidence of clinical and/or biochemical androgen excess. In addition, exclusion criteria were also applied to the control group
Petta *et al.* (2017) DOI: 10.1371/ journal.pone.0186136	202	101	33.2 ±5.5, 34.9 ± 8.2	ESHRE/ASRM	Italian	Cohort; January 2005–December 2015	Italy; PCOS outpatient clinic	General Practitioner referral	They were part of an ongoing project aimed at assessing cardiovascular risk and liver damage in the general population, according to presence or absence of fatty liver. All women enrolled as controls had available an abdominal and pelvic ultrasound performed during the last year, aimed at seeing both liver and ovarian morphology
Rahmanpour *et al.* (2012) DOI: 10.1016/ j.jpag.2012.02.004	30	71	17.73 ± 1.01, 17.69 ± 1.29	NIH	Iranian	Cross-sectional; not stated	Iran; randomly selected PCOS members from previous study	Same study as PCOS	Randomly selected adolescents from normal adolescents of previous study
Ramos and Spritzer (2015) DOI: 10.1016/ j.gene.2015.01.012	199	99	22.7 ± 7.1	ESHRE/ASRM	93.9% Caucasian	Cross-sectional; 2009–2013	Brazil; Advertisement via local media	Same as PCOS	
Roe *et al.* (2013) DOI: 10.1016/ j.jpeds.2012.11.019	148	57	16.9 ± 1.9, 16.6 ± 2.5	AE-PCOS	73.7% Caucasian	Retrospective; 2008–2010	USA; Outpatient Gynaecology clinic	Not stated	

(continued)

Supplementary Table SIV Continued

Author (year) DOI	PCOS (n)	Control (n)	Age, mean ± SD	PCOS definition	PCOS ethnicity	Study design; period	Country; PCOS recruitment source	Control recruit-ment source	Control description
Santos et al. (2018) DOI: 10.1186/s12958-018-0355-9	191	100	22.89 ± 6.66, 25.18 ± 7.72		Not stated	Cross-sectional; not stated	Brazil; advertisement in local media	Same as PCOS	
Shi et al. (2013) DOI: 10.1016/j.ejogrb.2013.06.002	1016	1016	29.58 ± 3.69, 29.77 ± 4.16	ESHRE/ASRM	Chinese	Case–control; 2009–2011	China; outpatient reproductive clinic	Same clinic as PCOS	Regular ovulatory menstrual cycles (26–32 days), normal androgen levels and ovary morphology, who received IVF treatment because of fallopian tube occlusion, and were in general good health
Wang et al. (2019) DOI: 10.1080/09513590.2019.1597343	73	63	Not stated	ESHRE/ASRM	Not stated	Case–control; December 2014–July 2017	China; outpatient clinic at Department of Obstetrics and Gynaecology	Outpatient infertility clinic	
Woo et al. (2012) DOI: 10.1507/endocrj.ej12-0055	87	53	25.3 ± 5.0, 27.8 ± 4.7	ESHRE/ASRM	Korean	Cross-sectional; not stated	South Korea; Gynaecology outpatient clinic at Hospital	Advertisements at the same hospital	Control subjects were women with regular menstrual cycles (25–35 days) and had no evidence of hirsutism, acne, alopecia or endocrine dysfunction, and no polycystic ovary (<12 follicles with size of 2–9 mm/ovary). Exclusion
Wu et al. (2018) DOI: 10.1080/09513590.2017.1336216	33	38	15.46 ± 1.01, 16.07 ± 1.14		Chinese	Retrospective case–control; January 2010–December 2013	China; outpatient Obstetrics and Gynaecology Department	Same clinic as PCOS	
					**Studies from Previous Systematic Review**				
Adali et al. (2008) DOI: 10.1177/147323000803600604	42	42	23.5 + 3.1	ESHRE/ASRM		Cross-sectional; September 2007–April 2008	Turkey, outpatients clinics of Department of Gynaecology and Obstetrics at University	Same clinic as PCOS	Age-matched, similar SES
Al-Ojaimi (2006) PMID: 16598331	134	479	29.4 + 5.5	ESHRE/ASRM		Cross-sectional; June 1996–June 2000	Bahrain, tertiary referral teaching hospital	Same department same time period	Normal menses, no hyperandrogenism
Altieri et al. (2010) DOI: 10.1016/j.ejogrb.2009.11.010	15	159	34.7 + 4.2	ESHRE/ASRM	Italian	Retrospective; January–April 2006	Italy, academic hospital	Same group as PCOS	Eumenorrhoeic, no signs of hyperandrogenism and normal ovarian morphology
Amato et al. (2008) DOI: 10.1111/j.1365-2265.2007.03145.x	170	34	24.5 + 5.8	ESHRE/ASRM		Retrospective, study period not stated	Italy, outpatients clinic	Same group as PCOS	Suspected of PCOS
Azziz et al. (2004a) DOI: 10.1210/jc.2003-031122	716	149	27.7 + 7.3	NIH		Cross-sectional, October 1987 and June 2002	USA, reproductive endocrinology clinic	Same group as PCOS	
Bernasconi et al. (1996) DOI: 10.1016/S0026-0495(96)90202-4	112	100	22.5 + 5.3; 23.2 + 6.9	ESHRE/ASRM		Cross-sectional; study period not stated	Italy, endocrinology clinic	Not stated	Nonhirsute, eumenorrhoeic, age matched

(continued)

Supplementary Table SIV Continued

Author (year) DOI	PCOS (n)	Control (n)	Age, mean ± SD	PCOS definition	PCOS ethnicity	Study design; period	Country; PCOS recruitment source	Control recruitment source	Control description
Beydoun *et al.* (2009) DOI: 10.1016/S1472-6483(10)60037-5	69	69	32.3 + 4.1	NIH		Retrospective; 1 January 2000–31 December 2006	USA, fertility treatment centre	Same time period and fertility treatment centre as PCOS	Age- and period-matched non-PCOS women
Carmina *et al.* (2006) DOI: 10.1210/jc.2005-1457	685	222	24.5 + 4.7; 24.3 + 4.5	ESHRE/ASRM		Retrospective, 1980–2004	Italy, two endocrinology departments at university	Same group as PCOS referred for hyperandrogenism	Idiopathic hirsutism and idiopathic hyperandrogenism; normal ovulatory cycles and normal ovaries on ultrasound
Chae *et al.* (2008) DOI: 10.1093/humrep/den239	143	255	25.5 + 5.8	ESHRE/ASRM	Korean	Retrospective; January 2004 to December 2007	South Korea, Department of Obstetrics and Gynaecology at Seoul National University Hospital	Healthcare centre in our hospital as a part of group check-up for work or an association or an individual need for annual comprehensive medical check-up with no specific health problems	No hirsutism, acne or male-type alopecia, all had regular menstrual cycles, none had PCO, none had any of the Rotterdam criteria
Chen *et al.* (2010) DOI: 10.1210/jc.2009-2698	273	278	24.5 + 5.1	ESHRE/ASRM		Cross-sectional, case-control; study period not stated	Taiwan, reproductive endocrinology clinic	Consecutive series (,35 years old) receiving voluntary annual medical check-up at the same hospital as PCOS	Matched by 3-year age strata
Cheung *et al.* (2008) DOI: 10.1093/humrep/den090	295	98	30.2 + 6.4	ESHRE/ASRM	Chinese	Cross-sectional; July 2003–April 2007	Hong Kong, endocrinology and infertility clinics at university hospital	61 from the community and the remaining were from same clinic as PCOS, mostly presented with tubal infertility	Regular menstrual cycles, no hirsutism/acne or ultrasound features of PCO
Chhabra and Venkatraman (2010) DOI: 10.3109/01443610903294154	100	95	15-34	NIH		Cross-sectional; study period not stated	India, gynaecological outpatient unit	From the same group	Menstrual dysfunction
Ciampelli *et al.* (2000) DOI: 10.1016/S0015-0282(99)00602-0	20	12	26.4 + 1.1; 27.3 + 1.0	ESHRE/ASRM		Cross-sectional; study period not stated	Italy, referred to authors' hospital	Not stated	Normo-ovulatory
Dokras *et al.* (2005) DOI: 10.1097/01.AOG.0000167408.30893.6b	129	177	Median; 28	NIH	96% White	Retrospective; 2002	USA, endocrinology clinic	Randomly selected from women who were seen for an annual examination by two healthcare providers in the gynaecology clinic over the same time period as the patients with PCOS.	Regular menses and an absence of hirsutism

(continued)

Supplementary Table SIV Continued

Author (year) DOI	PCOS (n)	Control (n)	Age, mean ± SD	PCOS definition	PCOS ethnicity	Study design; period	Country; PCOS recruitment source	Control recruitment source	Control description
Echiburú *et al.* (2008) DOI: 10.1016/ j.metabol.2008.08.002	159	93	24.3 + 5.8	NIH		Cross-sectional; 2002–2006	Chile, endocrinology unit of university	Community centres of the same geographical area as the patients had the same socioeconomic level.	Regular 28- to 32-day menstrual cycles, absence of hirsutism and other manifestations of hyperandrogenism, and absence of galactorrhoea and/or thyroid dysfunction
Economou *et al.* (2009) DOI: 10.14310/ horm.2002.1236	83	64	25.0 + 4.9	NIH		Cross-sectional; study period not stated	Greece, PCOS-endocrine unit at hospital	Not stated	Normo-androgenaemic and regularly ovulating women
Ferk *et al.* (2007) DOI: 10.1093/humrep/ del457	123	110	24.4 + 4.4	ESHRE/ASRM	European (Slovenian) origin	Cross-sectional; 2002–2005	Slovenia, Department of Obstetrics and Gynaecology at university	Authors' clinic	Age-matched, healthy with proven fertility (seen in clinic for normal pregnancy), no menstrual cycle irregularities, with no clinical or biochemical hyperandrogenism and without polycystic ovaries. They also had no history of endocrinological or auto-immune disorders and no surgery to the pelvic region
Glueck *et al.* (2003a) DOI: 10.1016/s0022-2143(03)00069-6	15	23	30.7 + 6.3	NIH	1 Black, 14 White	Cross-sectional; study period not stated	USA, possibly outpatients of cholesterol centre	Same group as PCOS, hospital personnel	Idiopathic intracranial hypertensive
Glueck *et al.* (2003b) DOI: 10.1016/s0026-0495(03)00104-5	138	1887	31.0 + 9.0	NIH	White	Cross-sectional; study period not stated	USA, location not stated	NHANES III	NHANES III
Glueck *et al.* (2005a) DOI: 10.1016/ j.lab.2004.09.011	37	28	PCOS and control: 34 median; IQR: 28–39	ESHRE/ASRM	PCOS and control: 64 White, 1 Black	Cross-sectional; study period not stated	USA, possibly outpatients of cholesterol centre	Same group as PCOS	Idiopathic intracranial hypertensive
Glueck *et al.* (2005b) DOI: 10.1016/ j.ejogrb.2005.03.010	401	2586	30.0 + 6.0	ESHRE/ASRM	Caucasian	Cross-sectional; study period not stated	USA, patients of cholesterol centre	NHANES I and community obstetrics practice study	Same age group
Glueck *et al.* (2006b) DOI: 10.1016/ j.metabol.2005.11.003	37	157	Median: 16.1	ESHRE/ASRM	White	Case-control; 1997–2003	USA, adolescents from Ohio, Kentucky, West Virginia, Indiana, Michigan, and included all at cholesterol centre	NHLBI growth and health study	Normal, regularly cycling
Glueck *et al.* (2008a) DOI: 10.1515/ jpem.2008.21.10.973	10	15	12.6 + 1.5	ESHRE/ASRM	2 Blacks 8 Whites	Cross-sectional; study period not stated	USA, outpatients of cholesterol centre	Same group as PCOS	
Glueck *et al.* (2009) DOI: 10.1016/ j.metabol.2009.02.005	488	261	Median: 35.2 (IQR 33–39)	ESHRE/ASRM	White	Case-control; July 1995–May 2008	USA, patients of cholesterol centre	Princeton follow-up study	Healthy free-living population, regular menses

(continued)

Supplementary Table SIV Continued

Author (year) DOI	PCOS (n)	Control (n)	Age, mean ± SD	PCOS definition	PCOS ethnicity	Study design; period	Country; PCOS recruitment source	Control recruitment source	Control description
Hahn *et al.* (2005) DOI: 10.1530/ eje.1.02024	120	50	29.0 + 5.4	NIH		Cross-sectional; study period not stated	Germany, outpatient clinics	Health-screening programme for employees instituted at the University of Duisburg-Essen Medical Centre and by public advertisement	No NIH PCOS or other medical conditions
Hahn *et al.* (2007) DOI: 10.1055/s-2007-967093	411	82	28 + 6.3	NIH	Caucasian	Cross-sectional; study period not stated	Germany, outpatient clinics	Screening programme for employees of the University of Duisburg-Essen	Matched in sociodemographic variables, including family status, education and employment. No PCOS (NIH) or any known medical condition
Liou *et al.* (2009) DOI: 10.1016/ j.fertnstert.2008.09.003	295	169	26.7 + 5.4	ESHRE/ASRM	Taiwan Chinese	Retrospective; 1 April 2004 to 31 December 2007	Taiwan, reproductive endocrinology clinic at Taipei Medical University	Same group as PCOS	No more than one of the following three PCOS components: (i) polycystic ovaries, (ii) oligomenorrhoea, and (iii) hyperandrogenism
Mukherjee *et al.* (2009) DOI: 10.1530/EJE-08-0932	180	144	24.8 + 5.3	ESHRE/ASRM	Indian	Case-control; study period not stated	India, infertility clinic and endocrinology clinic	General population	Age-matched, regular menstrual cycles and no clinical and/or biochemical signs of hyperandrogenaemia or polycystic ovaries
Nácul *et al.* (2007) DOI: 10.1016/ j.ejogrb.2006.09.009	31	21	22.4 + 7.1	NIH		Case-control; study period not stated	Brazil, consulting patients	Same group as PCOS	Age-matched women with regular, ovulatory cycles, normal androgen levels and idiopathic hirsutism
Pasquali *et al.* (1993) DOI: 10.1159/ 000182732	100	138	20.8 + 5.9	ESHRE/ASRM		Retrospective; study period not stated	Italy, location not stated	Those who attended the institute for evaluation and treatment of obesity or for general health check-up, and hospital staff	Normal menses, no hirsutism or other signs of androgenization
Patel *et al.* (2008) DOI: 10.4158/ ep.14.2.193	189	78	28.9 + 7.7	NIH	USA	Retrospective; September 2001–June 2006	USA, endocrinology clinic	Randomly selected from among female patient with regular menses by the same endocrinologist in the same time period	Regular menses recruited at same time period by the same endocrinologist
Shroff *et al.* (2007) DOI: 10.1016/ j.fertnstert.2007.01.032	258	110	27.0 + 5.3; 30.0 + 6.3; 28.9 + 5; 28.2 + 5.8	ESHRE/ASRM		Retrospective chart review; 2002–2005	USA, reproductive endocrinology clinic	Seen for annual examination at gynaecology clinic during the same time period	Regular menses, no hirsutism

(continued)

Supplementary Table SIV Continued

Author (year) DOI	PCOS (n)	Control (n)	Age, mean ± SD	PCOS definition	PCOS ethnicity	Study design; period	Country; PCOS recruitment source	Control recruitment source	Control description
Spranger *et al.* (2004) DOI: 10.1111/j.1365-2265.2004.02159.x	63	35	28.9 + 0.6	NIH		Cross-sectional; study period not stated	Germany, location not stated	Not stated	No menstrual disorders or signs of hyperandrogenism
Vrbikova *et al.* (2007) DOI: 10.1515/cclm.2007.113	244	57	27.4 + 7.5	ESHRE/ASRM		Cross-sectional; 1997–2006	Czech Republic, outpatient tertiary endocrine department	Via advertisement	All lacked symptoms of hyperandrogenism, had a regular menstrual cycle (21–35 days) and had androgen levels within the reference range
Wang *et al.* (2009) DOI: 10.1093/humrep/den430	271	296	28.9 + 3.4	ESHRE/ASRM	Chinese	Cross-sectional; 1 January 2004–30 August 2006	China, reproductive centre clinic	Same geographic area, recruited in the same period and evaluated consecutively	Age-matched, normal ovulatory menstrual cycles, absence of hirsutism and other manifestations of hyperandrogenism, and absence of sonographic signs of PCOS. None of them had sign of galactorrhoea and thyroid dysfunction or personal or family history of diabetes

Table design and previous 2012 SR contents taken from Lim *et al.* (2012) (*Human Reproduction Update*).
