# Supplementary material for: Childhood, adolescent, and adulthood adiposity are associated with risk of PCOS: a Mendelian randomization study with meta-analysis
Source: Hum Reprod. 2023 Apr 4;38(6):1168–82. doi: 10.1093/humrep/dead053 (PMC10233304; doi:10.1093/humrep/dead053)
Supplement: dead053_Supplementary_Table_SV [file dead053_supplementary_table_sv.pdf]

Human Reproduction, pp. 1–9, 2023
https://doi.org/10.1093/humrep/dead053
human reproduction
SUPPLEMENTARY DATA

Supplementary Table SV Additional details of included studies.

Author (year)	Country, PCOS recruitment source	Control recruitment source	Control description	Measurement of height, weight, waist, and/or waist–hip ratio	Diet and physical activity	Medications
			**Studies from Updated Systematic Review**			
Aarestrup *et al.* (2021)	Denmark; discharge diagnoses obtained by linked to the Danish National Patient Registry	Copenhagen School Health Records Registry	All children who attended a public or private school in Copenhagen			
Altinkaya *et al.* (2014)	Turkey; not stated	Not stated	Age-matched women who had regular menses and no clinical or biochemical hyperandrogenism or PCO		No diet change within last 3 months	No subject has been on hormonal contraceptives or other medications for three months prior to the study
Ates *et al.* (2018)	Turkey; Department of Gynaecology and Obstetrics at University	Same group as PCOS	Age-matched adolescent girls with regular menstrual cycles, normal ovaries on ultrasonography and the absence of symptoms of hyperandrogenism who were admitted to the clinic for other gynaecologic disorders	WC was measured as the smallest circumference at the level of the umbilicus and hip circumference was measured at the levels of the major trochanters through the pubic symphysis		
Ayonrinde *et al.* (2016)	Australia; Western Australian Pregnancy Cohort	Same group as PCOS				
Behboudi-Gandevani *et al.* (2017)	Iran; Outpatient Endocrinology Unit	Not stated	Healthy eumenorrhoeic non-hirsute women aged 20–40 years	The waist circumference (WC) was measured between the lower rib margin and the iliac crest at the end of a normal expiration. The hip circumference (HC) was measured at the levels of buttocks' maximum extension		Without any medication that interfered with the normal function of the hypothalamic–pituitary–gonadal axis such as hormones, glucocorticoids, insulin sensitizers, and anti-androgen therapies
Boyle *et al.* (2015)	Australia; Darwin Region Urban Indigenous Diabetes study participants—living in defined geographical region within Darwin area, Australia, for ≥6 months	Same group as PCOS	Non-hyperandrogenic or hyperandogenic or idiopathic hirsutism			
Carmina *et al.* (2019)	Italy; Department of Health Sciences at University	Same group as Control	Normal ovulatory women were selected as controls. These women were drawn from the same population base, had no complaints related to androgen excess and were matched for age with our patient population of women with PCOS			No patient had received any medication for at least 3 months before the study
Christensen *et al.* (2013)	USA; subset of patients enrolled in large population-based cohort study, KPSC Children's Health Study 2007–2009. This actively enrolled children in a large, prepaid integrated managed healthcare system	Same group as PCOS	No indication of NIH criteria			

(continued)

Supplementary Table SV Continued

Author (year)	Country, PCOS recruitment source	Control recruitment source	Control description	Measurement of height, weight, waist, and/or waist–hip ratio	Diet and physical activity	Medications
Çinar *et al.* (2016)	Turkey; outpatient adolescence clinic in tertiary referral hospital	Not stated	Normal menstrual cycles, with no evidence of hyperandrogenism and normal ovarian morphology on pelvic ultrasonography with a Ferriman–Gallwey score (FGS) <8	The BMI and WHR were assessed by a single investigator for all subjects		No subjects were treated with corticosteroids, or any other medication for at least 3 months before study participation statins aspirin, oral contraceptives
Dadachanji *et al.* (2015)	India; Infertility and Endocrinology outpatient clinic	General population	Age-matched regularly menstruating women with absence of any clinical and/or biochemical signs of hyperandrogenaemia and having normal ovaries on ultrasound imaging, with the same ethnic background			Participants who had taken any medication that would alter hormonal parameters or lipid and carbohydrate metabolism were excluded
De Medeiros *et al.* (2014)	Brazil; outpatient reproductive and endocrine clinic in a University Hospital	Same clinic as PCOS	Tubal or male cause of infertility and lack of PCOS	Weighed on an electronic scale, and their height was measured using a Harpender stadiometer (Holtain Limited, Crymych, Dyfed, UK). The waist was measured at the midway point between the lower rib margin and the iliac crest, and the hip was measured from the widest circumference over the great trochanters		
de Zegher *et al.* (2017)	Spain/Germany; University Hospitals in Barcelona, Spain and Datteln, Germany	Recruited from nearby schools and from hospital staff	Healthy, regular menses and had an ethnic background similar to those of PCOS	Height and weight at PCOS diagnosis were retrieved from medical records		
Fan *et al.* (2012)	China; outpatient reproductive endocrinology department at University Hospital	Same clinic as PCOS	Infertile women due to salpingian obstruction or the husband's infertility. All were clinically healthy women who had regular menstrual cycles (<35 days), exhibited normal circulating androgen concentrations and the absence of obvious acne or hirsutism on physical examination or polycystic ovaries as determined by ultrasound	Waist circumference was obtained as the smallest circumference at the level of the umbilicus. Hip circumference was obtained as the widest circumference at the level of the buttocks and waist-to-hip ratio was calculated to assess body-fat distribution. Body weight in light clothing and barefoot height were measured		
Gümüş *et al.* (2015)	Turkey; outpatient adolescence department	Same clinic as PCOS	Patients without PCOS who had been admitted for vaginal and urinary system infections			
Gourgari *et al.* (2015)	USA: Paediatric Endocrine Division of Infants and Children's Hospital and also from other physicians	Database at Healthy Volunteer Office of the National Institutes of Health + local advertisement	18–29 years old with normal menstrual function and without evidence of PCOS were eligible; they had to be off oral contraceptive pills or any other medications that alter steroidogenesis for at least 1 month prior to participating	The waist-to-hip was measured by our dietitian in all participants. Waist circumference was measured at the minimum circumference between the iliac crest and the rib cage. Hip circumference was measured at the maximum protuberance of the buttocks		

(continued)

Supplementary Table SV Continued

Author (year)	Country, PCOS recruitment source	Control recruitment source	Control description	Measurement of height, weight, waist, and/or waist–hip ratio	Diet and physical activity	Medications
Haldar et al. (2018)	India; Outpatient Department of Obstetrics and Gynaecology	Same clinic as PCOS	Age-matched healthy female volunteers with regular menstrual cycles (26–34 days), normal ovarian morphology, and normal total testosterone levels			
Hart et al. (2011	Australia; Western Australian Pregnancy Cohort (Raine) Study	Same cohort as PCOS				
Hickey et al. (2011)	Australia; Western Australian Pregnancy Cohort (Raine) Study	Same cohort as PCOS				Not taking any sex steroid hormones or other medication likely to interfere with pituitary, ovarian or endometrial function. Participants
Hudecova et al. (2011)	Sweden; out-patient register at University Hospital	Population registers	Residing in same county and born during the same month as the index patients. Healthy control status was assured by the absence of polycystic ovaries on transvaginal ultrasound. Furthermore, all control subjects denied a prior history of oligomenorrhoea or amenorrhoea (lasting more than 3 months)			
Kaewnin et al. (2018)	Thailand; University students in Bangkok	Same group as PCOS				
Kim et al. (2019)	Korea; PCOS speciality clinic at University Hospital	Healthcare centre in the same University Hospital	No specific age criteria. They had regular (21–35 days) menstrual cycles, a modified Ferriman-Gallwey score < 6, and no polycystic ovary morphology on pelvic ultra- sound examination. They were taking no medications and did not have physician-diagnosed diabetes			For patients on oral contraceptives at their evaluation, medication was discontinued for a minimum of 1 month before blood collection
Koivuaho et al. (2019)	Finland; Northern Finland Birth Cohort 1966	Same cohort as PCOS	Women without any PCOS symptoms at age 31 and without PCOS diagnosis by age 46			
Kyrkou et al. (2016)	Greece; outpatient clinic at Department of Gynaecology and Obstetrics	Healthcare professionals	Healthy volunteer females (students, medical and paramedical students, nurses, and doctors), who had normal ovulatory cycles (26–35 days) and no sign of hyperandrogenism			Treatment with compounds affecting sex hormones (oral contraceptives) or other medications within the previous 6 months served as exclusion criteria
Li et al. (2013)	China; outpatient reproductive clinic	Not stated	Regular menstrual cycle (26–35 days) and normal ovarian morphology. Total testosterone and modified Ferriman-Gallwey score were evaluated for exclusion of hyperandrogenism	Waist circumference was measured midway between the lowest rib and the iliac crest. Hip circumference was the longest measurement of hip		No hormonal therapy for at least 3 months prior to the test. Medication history were collected to exclude those who have had weight or glucose affecting measures like metformin or oral hypoglycaemic agents

(continued)

Supplementary Table SV Continued

Author (year)	Country, PCOS recruitment source	Control recruitment source	Control description	Measurement of height, weight, waist, and/or waist–hip ratio	Diet and physical activity	Medications
Nambiar *et al.* (2016)	India; women's health and fertility outpatient clinic	Same clinic as PCOS	Not stated			
Oztas *et al.* (2016)	Turkey; outpatient reproductive endocrinology clinic	Not stated	Healthy age-matched adolescents were also recruited as control group. All adolescents were further evaluated by an experienced, single paediatrician in order to exclude any possible concomitant endocrinological and other systemic diseases. All			Exclusion criteria included medications known to alter insulin secretion or action
Pepene (2012)	Romania; not stated	Not stated	Controls had no evidence of clinical and/or biochemical androgen excess. In addition, exclusion criteria were also applied to the control group			Exclusion based on current or previous use (within 6 months) of oral contraceptives, antiandrogens, infertility medication or drugs known to affect carbohydrate–lipid metabolism
Petta *et al.* (2017)	Italy; PCOS outpatient clinic	General Practitioner referral	They were part of an ongoing project aimed at assessing cardiovascular risk and liver damage in the general population, according to presence or absence of fatty liver. All women enrolled as controls had available an abdominal and pelvic ultrasound performed during the last year, aimed at seeing both liver and ovarian morphology	Waist circumference (WC) was measured at the midpoint between the lower border of the rib cage and the iliac crest		Excluded women treated with clomiphene citrate, oral contraceptives, antiandrogens, drugs to control their appetite or insulin-sensitizing drugs during the 6 months before the examination
Rahmanpour *et al.* (2012)	Iran; randomly selected PCOS members from previous study	Same study as PCOS	Randomly selected adolescents from normal adolescents of previous study	Waist circumference was measured midway between the lowest rib and the iliac crest with the subject standing at the end of gentle expiration, and hip circumference at the greater trochanters. Weight was measured in kilograms and height was measured to the nearest 0.5 cm		The exclusion criteria were, use of any medications known to affect sex hormone, glucose or lipid metabolism for at least a month and oral contraceptives for 3 months before entering the study
Ramos and Spritzer (2015)	Brazil; advertisement via local media	Same as PCOS				No participant received any drugs known to interfere with hormone levels (such as oral contraceptive pills, antiandrogens, metformin, fibrates, or statins) for at least 3 months before the study

(continued)

Supplementary Table SV Continued

Author (year)	Country, PCOS recruitment source	Control recruitment source	Control description	Measurement of height, weight, waist, and/or waist–hip ratio	Diet and physical activity	Medications
Roe et al. (2013)	USA; Outpatient Gynaecology clinic	Not stated				
Santos et al. (2018)	Brazil; advertisement in local media	Same as PCOS				Exclusion criteria included having used drugs known to interfere with hormone levels (such as oral contraceptive pills, antiandrogens, progestins, metformin, fibrates, or statins) for 3 or more months before the study
Shi et al. (2013)	China; outpatient reproductive clinic	Same clinic as PCOS	Regular ovulatory menstrual cycles (26–32 days), normal androgen levels and ovary morphology, who received IVF treatment because of fallopian tube occlusion, and were in general good health			
Wang et al. (2019)	China; outpatient clinic at Department of Obstetrics and Gynaecology	Outpatient infertility clinic				
Woo et al. (2012)	South Korea; Gynaecology outpatient clinic at Hospital	Advertisements at the same hospital	Control subjects were women with regular menstrual cycles (25-35 days) and had no evidence of hirsutism, acne, alopecia or endocrine dysfunction, and no polycystic ovary (<12 follicles with size of 2–9 mm/ovary). Exclusion			None of the subjects had taken any medications for at least 6 months prior to the study, including oral contraceptives, glucocorticoids, ovulation induction agents, anti-obesity drugs, and estrogenic, anti-androgenic, or anti-hypertensive medication
Wu et al. (2018)	China; Outpatient Obstetrics and Gynaecology Department	Same clinic as PCOS				
			**Studies from Previous Systematic Review**			
Adali et al. (2008)	Turkey; Outpatients clinics of Department of Gynaecology and Obstetrics at University	Same clinic as PCOS	Age-matched, similar SES			No oral contraceptives, glucocorticoids, antiandrogens, insulin sensitizers, or psychiatric medications for 6 months prior
Al-Ojaimi (2006)	Bahrain, tertiary referral teaching hospital	Same department same time period	Normal menses, no hyperandrogenism			
Altieri et al. (2010)	Italy, academic hospital	Same group as PCOS	Eumenorrhoeic, no signs of hyperandrogenism, and normal ovarian morphology			
Amato et al. (2008)	Italy, outpatients clinic	Same group as PCOS	Suspected of PCOS			No clomiphene citrate, oral contraceptives, anti-androgens, drugs to control their appetite, or insulin sensitizing drugs (metformin, pioglitazone, rosiglitazone) 6 months prior

(continued)

Supplementary Table SV Continued

Author (year)	Country, PCOS recruitment source	Control recruitment source	Control description	Measurement of height, weight, waist, and/or waist–hip ratio	Diet and physical activity	Medications
Azziz et al. (2004b)	USA, reproductive endocrinology clinic	Same group as PCOS				
Bernasconi et al. (1996)	Italy, endocrinology clinic	Not stated	Nonhirsute, eumenorrhoeic, age-matched			No medications for hirsutism for 3 months prior
Beydoun et al. (2009)	USA, fertility treatment centre	Same time period and fertility treatment centre as PCOS	Age- and period-matched non PCOS women			
Carmina et al. (2006)	Italy, two endocrinology departments at university	Same group as PCOS-referred for hyperandrogenism	Idiopathic hirsutism and idiopathic hyperandrogenism; normal ovulatory cycles and normal ovaries on ultrasound			No medications for 3 months prior
Chae et al. (2008)	South Korea, Department of Obstetrics and Gynaecology at Seoul National University Hospital	Healthcare centre in our hospital as a part of group checkup for work or an association or an individual need for annual comprehensive medical checkup with no specific health problems	No hirsutism, acne or male-type alopecia, all had regular menstrual cycles, none had PCO, none had any of the Rotterdam criteria			No medication affecting gonadotrophin status for 6 months prior
Chen et al. (2010)	Taiwan, reproductive endocrinology clinic	Consecutive series (<35 years old) receiving voluntary annual medical check-up at the same hospital as PCOS	Matched by 3-year age strata			No use of oral contraceptives within 6 months of enrolment, or the use of medications known to affect the hypothalamic–pituitary–ovarian axis, such as anti-androgens, ovulation induction agents, antidiabetic medications, anti-obesity medications, or glucocorticoids
Cheung et al. (2008)	Hong Kong, endocrinology and infertility clinics at university hospital	61 from the community and the remaining were from same clinic as PCOS, mostly presented with tubal infertility	Regular menstrual cycles, no hirsutism/acne, or ultrasound features of PCO	Narrowest measurement midway between the top of the iliac crest and the lower rib margin, whereas the hip circumference was taken as the widest measurement at the level of the greater trochanters		No use of medications known to affect steroid or glucose metabolism in past 3 months
Chhabra and Venkatraman (2010)	India, gynaecological outpatient unit	From the same group	Menstrual dysfunction			
Ciampelli et al. (2000)	Italy, referred to authors' hospital	Not stated	Normo-ovulatory			No medication known to affect plasma sex steroid levels for at least 3 months prior
Dokras et al. (2005)	USA, endocrinology clinic	Randomly selected from women who were seen for an annual examination by two healthcare providers in the gynaecology	Regular menses and an absence of hirsutism			3/129 using cholesterol-lowering medications

(continued)

Supplementary Table SV Continued

Author (year)	Country, PCOS recruitment source	Control recruitment source	Control description	Measurement of height, weight, waist, and/or waist–hip ratio	Diet and physical activity	Medications
		clinic over the same time period as the patients with PCOS				
Echiburú *et al.* (2008)	Chile, endocrinology unit of university	Community centres of the same geographical area as the patients and had the same socioeconomic level	Regular 28- to 32-day menstrual cycles, absence of hirsutism and other manifestations of hyperandrogenism, and absence of galactorrhoea and/or thyroid dysfunction	Height-nearest 0.1 cm using wall-amounted stadiometer; weight-nearest 0.1 kg using a hospital balance beam scale		
Economou *et al.* (2009)	Greece, PCOS-endocrine unit at hospital	Not stated	Normo-androgenaemic and regularly ovulating women		Excluded if they have a history of alcohol intake of higher than 20 g per day	No oral contraceptives, insulin sensitizers or any medication interfering with metabolic or hormonal measurements in 3 months prior
Ferk *et al.* (2007	Slovenia, Department of Obstetrics and Gynaecology at university	Authors' clinic	Age-matched, healthy with proven fertility (seen in clinic for normal pregnancy), no menstrual cycle irregularities, with no clinical or biochemical hyperandrogenism and without PCO. They also had no history of endocrinological or auto- immune disorders and no surgery to the pelvic region			
Glueck *et al.* (2003)	USA, possibly outpatients of cholesterol centre			Without shoes or over-clothes		No hormones, corticosteroids, or anticoagulants
Glueck *et al.* (2003)	USA, possibly outpatients of cholesterol centre	Same group as PCOS, hospital personnel	Idiopathic intracranial hypertensive			
Glueck *et al.* (2005a)	USA, patients of cholesterol centre	NHANES I and community obstetrics practice study	Same age group	Clothed weight		No androgens, oestrogen-progestins, oestrogen-androgens, or drugs known to effect endogenous sec hormones or lipoprotein metabolism in 2 months prior
Glueck *et al.* (2005b)	USA, possibly outpatients of cholesterol centre	Same group as PCOS	Idiopathic intracranial hypertensive			
Glueck *et al.* (2006)	USA, adolescents from Ohio, Kentucky, West Virginia, Indiana, Michigan, and included all at cholesterol centre	NHLBI Growth and Health Study	Normal, regularly cycling			No oestrogen-progestin oral contraceptives
Glueck *et al.* (2008)	USA, patients of cholesterol centre				Encouraged to stay on low-carbohydrate high-protein diet	

(continued)

Supplementary Table SV Continued

Author (year)	Country, PCOS recruitment source	Control recruitment source	Control description	Measurement of height, weight, waist, and/or waist–hip ratio	Diet and physical activity	Medications
Glueck *et al.* (2009)	USA, patients of cholesterol centre	Princeton follow-up study	Healthy free-living population, regular menses	Light indoor clothing without shoes, two measurements, if differed by more than 0.5 cm for ht or 0.3 kg for wt, third measurement will be done		
Hahn *et al.* (2005)	Germany, outpatient clinics	Health-screening programme for employees instituted at the University of Duisburg-Essen Medical Centre and by public advertisement	No NIH-PCOS or other medical conditions			No prescription medication except allergy medications and occasional pain medications for at least 3 months
Hahn *et al.* (2007)	Germany, outpatient clinics	Screening programme for employees of the University of Duisburg-Essen	Matched in sociodemographic variables, including family status, education, and employment. No PCOS (NIH) or any known medical condition			No medication known to affect carbohydrate and lipid metabolism or endocrine parameters for at least 3 months
Liou *et al.* (2009)	Taiwan, reproductive endocrinology clinic at Taipei Medical University	Same group as PCOS	No more than one of the following three PCOS components: (i) PCOM, (ii) Oligo-An, and (iii) HA			
Mukherjee *et al.* (2009)	India, infertility clinic and endocrinology clinic	General population	Age-matched, regular menstrual cycles and no clinical and/or biochemical signs of hyperandrogenaemia or polycystic ovaries			No medication known to affect carbohydrate and lipid metabolism or endocrine parameters for 3 months prior
Nácul *et al.* (2007)	Brazil, consulting patients (from where?)	Same group as PCOS	Age-matched women with regular, ovulatory cycles, normal androgen levels, and idiopathic hirsutism			No drugs known to interfere with hormonal levels for 3 months prior; no heparin or aspirin within 15 days of test
Pasquali *et al.* (1993)	Italy, location not stated	Those who attended the institute for evaluation and treatment of obesity or for general health checkup, and hospital staff	Normal menses, no hirsutism, or other signs of androgenization	Without shoes to the nearest 0.5 cm and body weight without clothes		
Patel *et al.* (2008)	USA, endocrinology clinic	Randomly selected from among female patients with regular menses by the same endocrinologist in the same time period	Regular menses recruited at same time period by the same endocrinologist			No lipid powering agents or anti-hypertensive agents (except spironolactone)
Shroff *et al.* (2007)	USA, reproductive endocrinology clinic	Seen for annual examination at gynaecology clinic during same time period	Regular menses, no hirsutism			
Spranger *et al.* (2004)	Germany, location not stated	Not stated	No menstrual disorders or signs of hyperandrogenism			No medication

(continued)

Supplementary Table SV Continued

Author (year)	Country, PCOS recruitment source	Control recruitment source	Control description	Measurement of height, weight, waist, and/or waist–hip ratio	Diet and physical activity	Medications
Vrbikova *et al.* (2007)	Czech Republic, outpatient tertiary endocrine department	Via advertisement	All lacked symptoms of hyperandrogenism, had a regular menstrual cycle (21–35 days), and had androgen levels within the reference range	To nearest 0.1 kg and height to nearest cm		
Wang *et al.* (2009)	China, reproductive centre clinic	Same geographic area, recruited in the same period and evaluated consecutively	Age-matched, normal ovulatory menstrual cycles, absence of hirsutism and other manifestations of hyperandrogenism, and absence of sonographic signs of PCOS. None of them had sign of galactorrhoea and thyroid dysfunction or personal or family history of diabetes			No hormonal preparations, including oral contraceptives for at least 3 weeks prior

Table design and previous SR contents taken from 2012 SR by Lim *et al.* (2012) (*Human Reproduction Update*).
