# Supplementary material for: Childhood, adolescent, and adulthood adiposity are associated with risk of PCOS: a Mendelian randomization study with meta-analysis
Source: Hum Reprod. 2023 Apr 4;38(6):1168–82. doi: 10.1093/humrep/dead053 (PMC10233304; doi:10.1093/humrep/dead053)
Supplement: dead053_Supplementary_Table_SVI [file dead053_supplementary_table_svi.pdf]

Human Reproduction, pp. 1–2, 2023
https://doi.org/10.1093/humrep/dead053

human reproduction

SUPPLEMENTARY DATA

Supplementary Table SVI Summary of quality assessment of included studies.

Author (year)	Selection	Comparability	Exposure	Total
Aarestrup et al. (2021)	2	1	2	5
Adali et al. (2008)	2	2	2	6
Al-Ojaimi (2006)	2	1	2	5
Altieri et al. (2010)	2	0	1	3
Altinkaya et al (2014)	3	1	1	5
Amato et al. (2008)	2	1	1	4
Ates et al. (2018)	3	2	2	7
Ayonrinde et al. (2016)	3	0	2	5
Azziz et al. (2004a)	2	0	2	4
Behboudi-Gandevani et al. (2017)	3	1	2	6
Bernasconi et al. (1996)	2	2	0	4
Beydoun et al. (2009)	2	2	1	5
Boyle et al. (2015)	2	1	1	4
Carmina et al. (2019)	3	2	2	7
Carmina et al. (2006)	2	0	1	3
Chae et al. (2008)	3	1	2	6
Chen et al. (2010)	2	1	0	3
Cheung et al. (2008)	3	0	2	5
Chhabra and Venkatraman (2010)	2	0	1	3
Christensen et al. (2013)	2	1	2	5
Ciampelli et al. (2000)	2	0	0	2
Çinar et al. (2016)	3	1	2	6
Dadachanji et al. (2015)	3	2	3	8
De Medeiros et al. (2014)	2	0	2	4
de Zegher et al. (2017)	2	1	3	6
Dokras et al. (2005)	2	1	0	3
Echiburú et al. (2008)	3	1	2	6
Economou et al. (2009)	2	0	2	4
Fan et al. (2012)	2	1	2	5
Ferk et al. (2007)	3	2	0	5
Glueck et al. (2003a)	2	0	0	2
Glueck et al. (2003b)	3	0	1	4
Glueck et al. (2005a)	3	1	1	5
Glueck et al. (2005b)	2	2	0	4
Glueck et al. (2006b)	3	1	1	5
Glueck et al. (2008a)	2	0	2	4
Glueck et al. (2009)	3	1	1	5
Gourgari et al. (2015)	2	1	2	5
Gümüş et al. (2015)	2	1	2	5
Hahn et al. (2005)	3	0	2	5
Hahn et al. (2007)	3	2	2	7
Haldar et al. (2018)	3	1	2	6

(continued)

Supplementary Table SVI Continued

Author (year)	Selection	Comparability	Exposure	Total
Hart *et al.* (2011)	2	1	2	5
Hickey *et al.* (2011)	3	1	2	6
Hudecova *et al.* (2011)	3	2	0	5
Kaewnin *et al.* (2018)	1	0	2	3
Kim *et al.* (2019)	4	2	2	8
Koivuaho *et al.* (2019)	1	0	2	3
Kyrkou *et al.* (2016)	2	1	2	5
Li *et al.* (2013)	2	0	3	5
Liou *et al.* (2009)	2	0	1	3
Mukherjee *et al.* (2009)	4	1	2	7
Nácul *et al.* (2007)	2	1	2	5
Nambiar *et al.* (2016)	2	0	2	4
Oztas *et al.* (2016)	2	1	3	6
Pasquali *et al.* (1993)	2	1	2	5
Patel *et al.* (2008)	2	1	1	4
Pepene (2012)	1	0	3	4
Petta *et al.* (2017)	4	1	3	8
Rahmanpour *et al.* (2012)	2	1	3	6
Ramos and Spritzer (2015)	3	0	2	5
Roe *et al.* (2013)	1	0	1	2
Santos *et al.* (2018)	2	0	2	4
Shi *et al.* (2013)	2	1	2	5
Shroff *et al.* (2007)	1	1	0	2
Spranger *et al.* (2004)	2	0	0	2
Vrbikova *et al.* (2007)	3	0	2	5
Wang *et al.* (2019)	2	1	3	6
Wang *et al.* (2009)	4	2	2	8
Woo *et al.* (2012)	2	0	3	5
Wu *et al.* (2018)	2	1	3	6

Maximum score of 9.
SELECTION.
(1) Is the definition of PCOS adequate? (a) yes, with independent validation*, (b) yes, e.g. record linkage or based on self-reports, (c) no description.
(2) Representativeness of the cases: (a) consecutive or obviously representative series of cases*, (b) potential for selection biases or not stated.
(3) Selection of controls: (a) community controls*, (b) hospital controls, (c) no description.
(4) Definition of controls: (a) no feature of PCOS*, (b) no description of source.
COMPARABILITY.
(1) Comparability of cases and controls on basis of design or analysis: (a) study controls for age*, (b) study controls for any additional factor*.
EXPOSURE.
(1) Ascertainment of exposure: (a) secure record (e.g. surgical records) or independently measured*, (b) structured interview where blind to case/control status*, (c) interview not blinded to case/control status, (d) written self-report or medical record only, (e) no description.
(2) Same method of ascertainment for cases and controls: (a) yes*, (b) no.
(3) Non-response rate: (a) same rate for both groups*, (b) non-respondents described, (c) rate different and no designation.
*A study can be awarded a maximum of one star for each numbered item within the Selection and Exposure categories. A maximum of two stars can be given for Comparability.
Table design and table legend taken from Lim *et al.* (2012) (*Human Reproduction Update*).
